# The Medicinal Chemistry in the Era of Machines and Automation: Recent Advances in Continuous Flow Technology

**DOI:** 10.1021/acs.jmedchem.9b01956

**Published:** 2020-02-12

**Authors:** Antimo Gioiello, Alessandro Piccinno, Anna Maria Lozza, Bruno Cerra

**Affiliations:** Laboratory of Medicinal and Advanced Synthetic Chemistry (Lab MASC), Department of Pharmaceutical Sciences, University of Perugia, Via del Liceo 1, 06123 Perugia, Italy

## Abstract

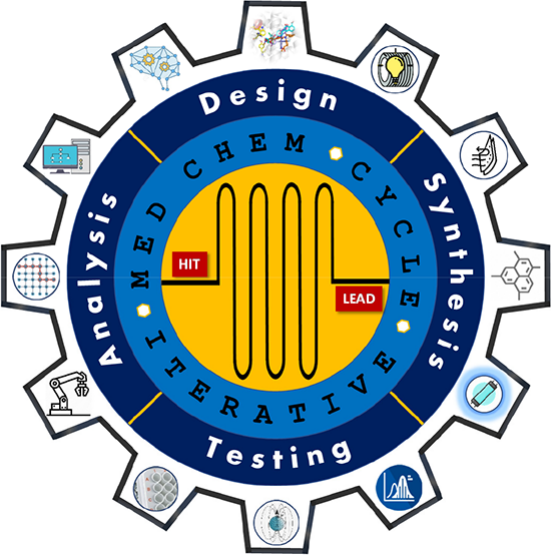

Medicinal chemistry plays a fundamental and underlying role in chemical biology, pharmacology, and medicine to discover safe and efficacious drugs. Small molecule medicinal chemistry relies on iterative learning cycles composed of compound design, synthesis, testing, and data analysis to provide new chemical probes and lead compounds for novel and druggable targets. Using traditional approaches, the time from hypothesis to obtaining the results can be protracted, thus limiting the number of compounds that can be advanced into clinical studies. This challenge can be tackled with the recourse of enabling technologies that are showing great potential in improving the drug discovery process. In this Perspective, we highlight recent developments toward innovative medicinal chemistry strategies based on continuous flow systems coupled with automation and bioassays. After a discussion of the aims and concepts, we describe equipment and representative examples of automated flow systems and end-to-end prototypes realized to expedite medicinal chemistry discovery cycles.

## 1 The Medicinal Chemistry (R)Evolution: Drawbacks and Technological Solutions

**Figure 1:**
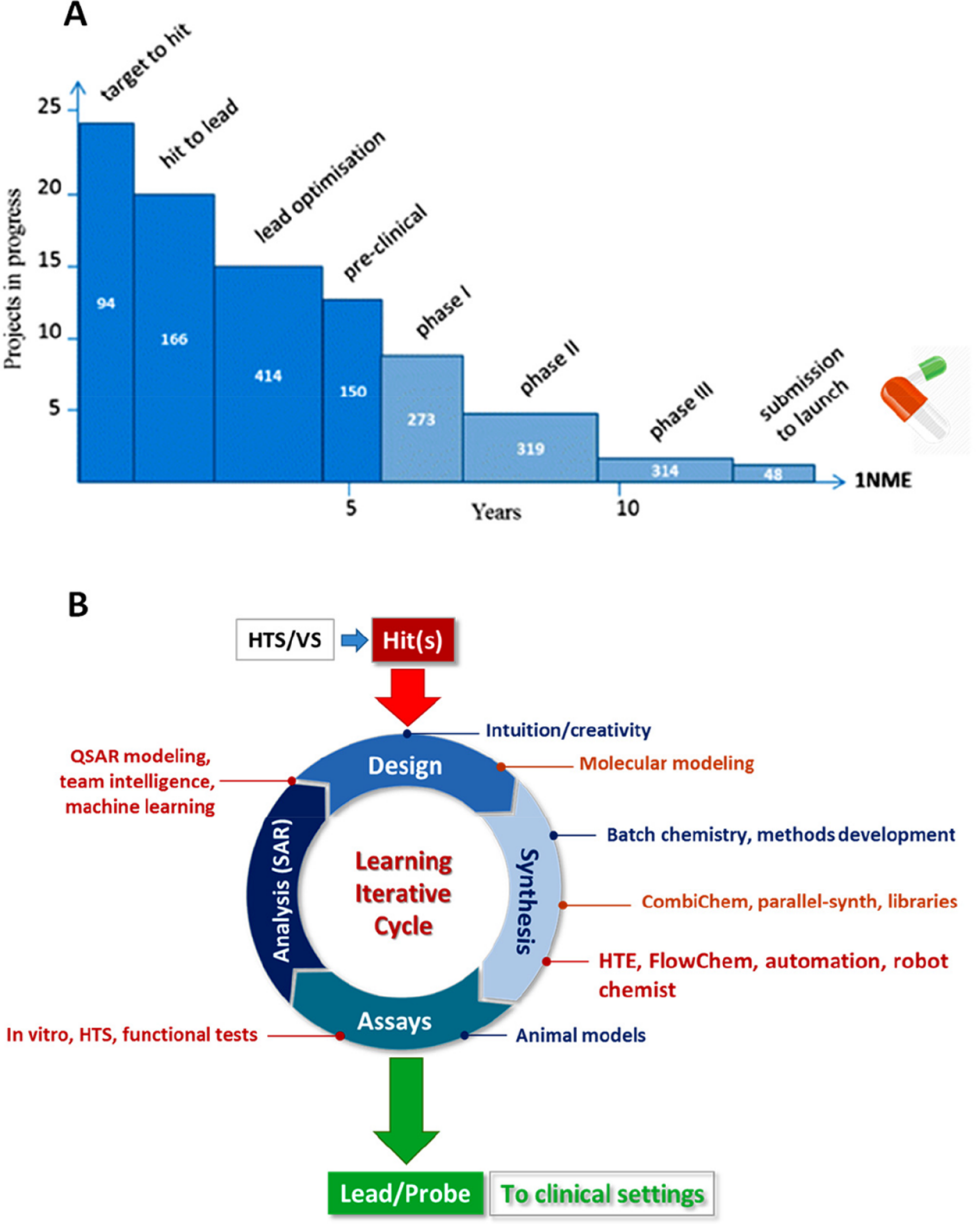
(A) Cost and timing in early stages of drug discovery. Adapted from ref (6). Copyright 2010 Nature Publishing Group. (B) Iterative learning cycles of medicinal chemistry based on diverse discipline activities with examples of key approaches used before 1980 (blue), up to 2000 (orange), and nowadays (red).

Medicinal chemistry is an interdisciplinary science at the interface of chemical biology, pharmacology, and medicine, playing a crucial role in drug discovery. The main objectives of medicinal chemistry are (i) to discover chemical probes and lead compounds for understudied biological targets, (ii) to demonstrate target druggability, and (iii) to address issues that determine drug success or failure.^1,2^ Most importantly, medicinal chemistry enables the identification of clinical candidates and provides novel strategies aimed at improving the range and quality of hit- and lead-finding phases that, although often underestimated, are critical to reduce attrition in drug discovery. Indeed, the majority of drug failures are due to the lack of efficacy and safety that can be related with the target and/or the chemical structure of the lead compound series.^3−5^ The selection of which lead series to explore is therefore a crux that can impact the rate of success in drug discovery. However, catching the right series from a number of possibilities remains difficult. Over the past three decades, numerous approaches have been proposed to solve this issue such as parallel chemical explorations based on combinatorial chemistry (CombiChem) and diversity oriented synthesis (DOS), and prediction models are increasingly accurate. Despite the progress made, identifying novel leads still remains an extremely complex and burdensome task in terms of time and costs. It has been estimated that for every drug approved, an average of about 20 hit-to-lead explorations and 15 lead optimization programs are required at a cost of around $600 million (32% of the overall cost to launch a novel drug) (Figure 1A).^6^ Not surprisingly, expediting early drug discovery is therefore a persisting issue that needs multimodal approaches and innovative solutions. Accordingly, pharmaceutical companies and academic groups are both engaged in new concepts and a deep (r)evolution in general thinking and strategies in response to new discoveries and technologies.^7−9^

Up to 1980, there was limited information regarding biological targets and their implications on disease mechanisms and hence their potential therapeutic applications.^10^ Because of the lack of rapid in vitro screening capabilities, compounds were designed and individually synthesized in gram quantities to satisfy the request of material for testing in animal models. Given the restricted methods and tools available at that time, these syntheses were often time-consuming, risky, and poorly efficient. The output encompassed few products per week, and compound libraries for lead identification were very limited. These drawbacks made the discovery process slow, and achievements were mainly obtained thanks to the intuition and creativity of researchers and, in some cases, by serendipitous findings. This chemistry-inspired/pharmacology-driven approach has evolved in a biology-inspired/technology-driven process. The advent of high-throughput screening (HTS), computational modeling, and, most recently, artificial intelligence (AI) and learning machines (ML), has enabled the rapid design and evaluation of a huge number of compounds.^11,12^ In vitro screening of in-house compounds collections and virtual screening campaigns can disclose diverse classes of active compounds (hits) that are chemically elaborated to furnish analogues with improved properties (leads). Having powerful synthetic capabilities in terms of compound throughput and scalability is therefore crucial to meet the constant demand for compounds to test. Despite the advances in combinatorial chemistry, parallel, and diversity-oriented synthesis, the synthesis of compound collections is still not sufficiently efficient and remains a bottleneck in the pace of early drug discovery.

Moreover, the iterative learning process of medicinal chemistry defining structure–activity relationships (SAR) is composed by computational design, compound synthesis, biological assays, and data collection whose analysis drives the next learning cycle (Figure 1B).^13^ Typically, cycle stages are compartmentalized, compounding delays from hypothesis to results, slow explorations, and a limited number of compounds for clinical trials. Strategies aimed at integrating the diverse disciplines and facilitating operations within the single compartment are therefore highly desirable.

In the past decade, flow-based platforms have emerged as an enabling technology that can solve such drawbacks. Continuous flow systems have demonstrated their potential in the rapid assembly of compound collections, in straightforward optimization and scale-up of relevant products and, more recently, being further applied to the development and manufacturing phase of active pharmaceutical ingredients (API).^14−16^ In early 2019, the IUPAC organization named flow chemistry among the top ten emerging technologies in chemistry^17^ and the FDA declared continuous manufacturing (CM) as one of the most important tool in the modernization of the pharmaceutical industry.^18^ Beyond the adoption for manufacturing innovation and novel sustainable methods, flow technology is increasingly exploited for medicinal chemistry projects. Indeed, flow chemistry can be applied to reach unexplored or inaccessible chemical space using traditional batch approaches to automate usual synthetic bench operations (as reagent loading, mixing, workup, purification, analysis, etc.) and accelerate library building, as well as to realize platforms for lead discovery. As we will discuss later, the integration of fluidics-assisted synthesize-and-test platforms coupled with automation and AI in molecular design, synthesis, and compound optimization bears the promise of making medicinal chemistry learning cycles more efficient.

As a complement to prior reviews^15,16,19−24^ dealing with the relevance of flow-based approaches in organic synthesis and method development, in this Perspective, we discuss contributions of eminent scientists on the development of automated flow systems and their impact on medicinal chemistry and drug discovery. We will focus on examples that have demonstrated the utility of flow technology for the automated synthesis of compound collections readily available for screenings as well as on closed-loop strategies highlighting the potential of self-standing platforms controlled by robots and machines. Concepts and integrative strategies and the description of flow equipment, analytical devices, tools for automation, and biological assays are also illustrated.

## 2 Automated Flow Systems to Power Medicinal Chemistry: Concepts and Equipment

Automation in drug discovery is not a new concept.^25^ Solid-phase peptide synthesis was made automated in the 1960s. Nowadays, automated HTS screening of compound libraries has become routine in both pharmaceutical companies and academic laboratories. Other applications include compound repositories, high-throughput experimentation (HTE), parallel/combinatorial synthesis, decision-making support systems, virtual screenings, and molecular design.^8^

Appropriate automation in chemistry and biology has become an important driver to innovate discovery processes while improving efficiency and reducing costs and timelines. Target screening against compound collections is relatively low cost, rapid, and extremely useful to identify hit compound series for optimization stages. However, the efficiency of HTS campaigns depends on compound availability and synthesis. The preparation of pure compound collections is often viewed as a limiting factor of medicinal chemistry, as bench chemistry is labor-intensive and time-consuming work. The automation, parallelization, and integration of chemical synthesis with purification and analysis is therefore essential to guarantee the constant and rapid supply of pure compounds ready for testing as well as to improve reproducibility and costs if compared with manual, serial compound synthesis.^8,26^

**Figure 2:**
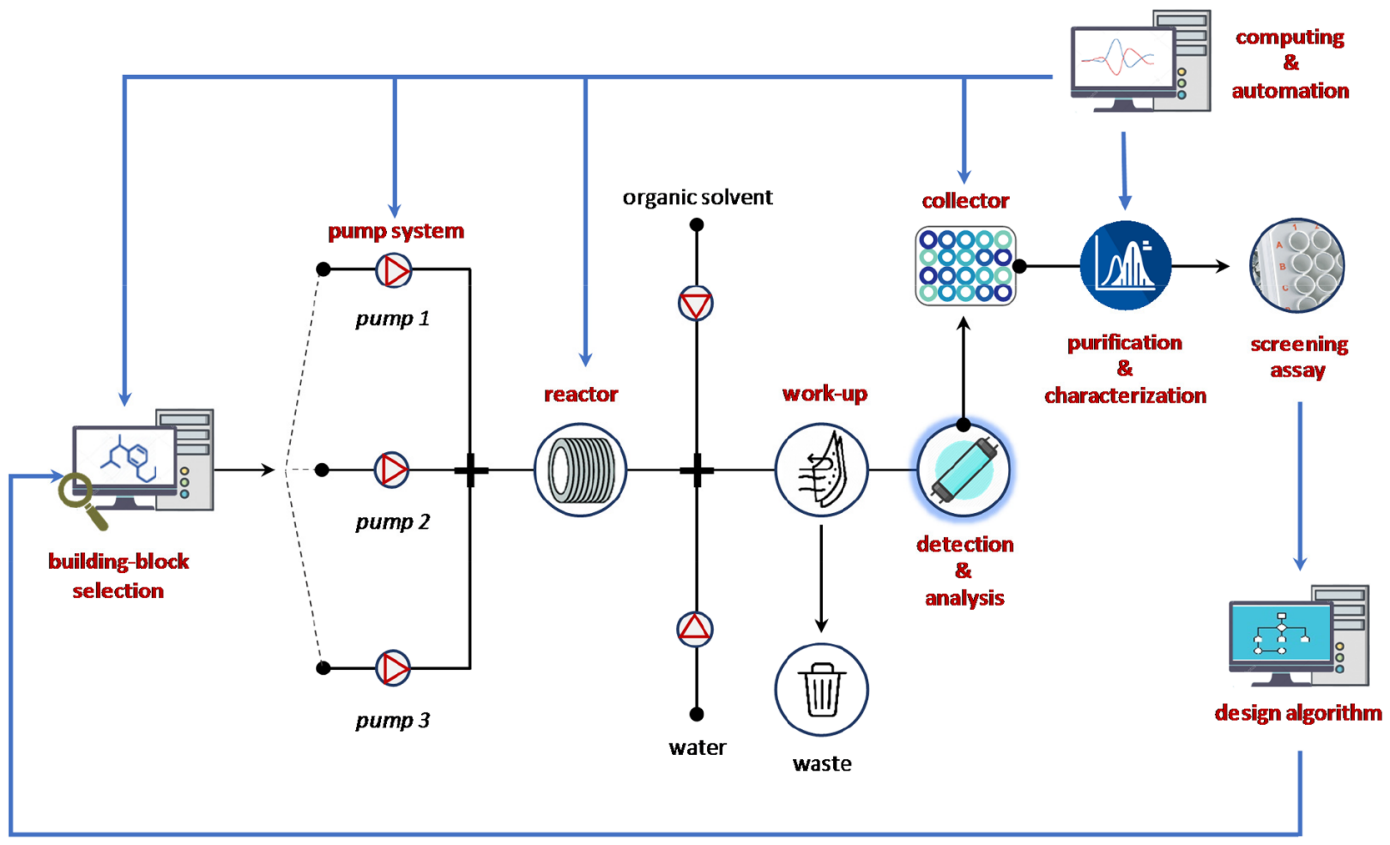
Integrated fluidic workflow for the automated molecular design–synthesis–screening–analysis–optimization for iterative medicinal chemistry discovery cycles.

Automation in synthesis and related technologies have therefore gained a central role in lead and drug discovery as they may offer solutions to overcome current limitations.^7,27,28^ To leverage the power of synthesis, a number of enabling chemical technologies exist that can facilitate the execution of chemical transformations and expedite compound synthesis. Among these, machine-assisted flow-based approaches have not only increased chemistries suitable for automation but also improved efficiency, safety, and environmental impact. Such approaches take advantage of automation, computer control, and robotics to limit manual and repetitive experimental operations and to increase time for creativity and innovation.^29−31^ Furthermore, integration of reaction steps with downstream processes, such as in-line software-assisted analytical devices, predictive computational tools, and feedback controls, can lead to streamlined synthesis and medicinal chemistry process (Figure 2).^32−34^ For example, the integration of synthesis platforms with ML and chemical artificial intelligence (CAI) is set to revolutionize the way in which chemists design and discover new molecules, especially if coupled with real-time screening.^11,12^ Although fascinating, the practical realization of fully integrated platforms remains very challenging and, at the moment, only within the reach of pharmaceutical companies and few leading academic research groups.

In the following paragraphs, we illustrate the diverse equipment that may be assembled to realize autonomous discovery systems and how a single device, or method, has been applied during the validation stage. The intent is not to be exhaustive but rather to provide readers with the background and state-of-the art of technologies that can complement flow synthesizers and power medicinal chemistry.

### 2.1 Flow Synthesizers

The use of flow synthesizers has been demonstrated to ideally complement or replace batch chemistry because of several advantages.^20^ First of all, flow synthesizers ensure a more accurate control over the reaction parameters (concentration, temperature, pressure, and reaction time) that can translate into higher product quality, robust methods, and hence into a smaller footprint in manufacturing plants. Flow reactions undergo to efficient mixing and heat/mass transfer with beneficial impact on reaction rates and productivity, while the pressurization of the devices allows operations at superheated conditions widening the reactivity window. Safety is an additional relevant aspect of flow synthesizers as they ensure the containment of hazardous or malodorous substances and the conduction of risky chemical transformations. Along this line, integration of flow synthesizers with downstream devices, automation and in-line reaction monitoring may further reduce manual handling and risks for operators, also for telescoped and multistep synthesis.^22^

Basic flow devices can be assembled by “do-it-yourself” approaches by recycling parts from HPLC and GC instrumentation (e.g., pumps or part of the pumps, connectors, tubing, injection valves, autosamplers, and fraction collectors).^35^ The use of computer-aided design (CAD) and 3D-printing techniques has now allowed the homemade building of customized mixing elements, residence time loops, separation units, chips, and reactors for specific flow applications.^36−38^ The upsurge of interest in continuous flow chemistry led also to relatively simple, user-friendly, and commercially available modular flow equipment.^20,39^

**Figure 3:**
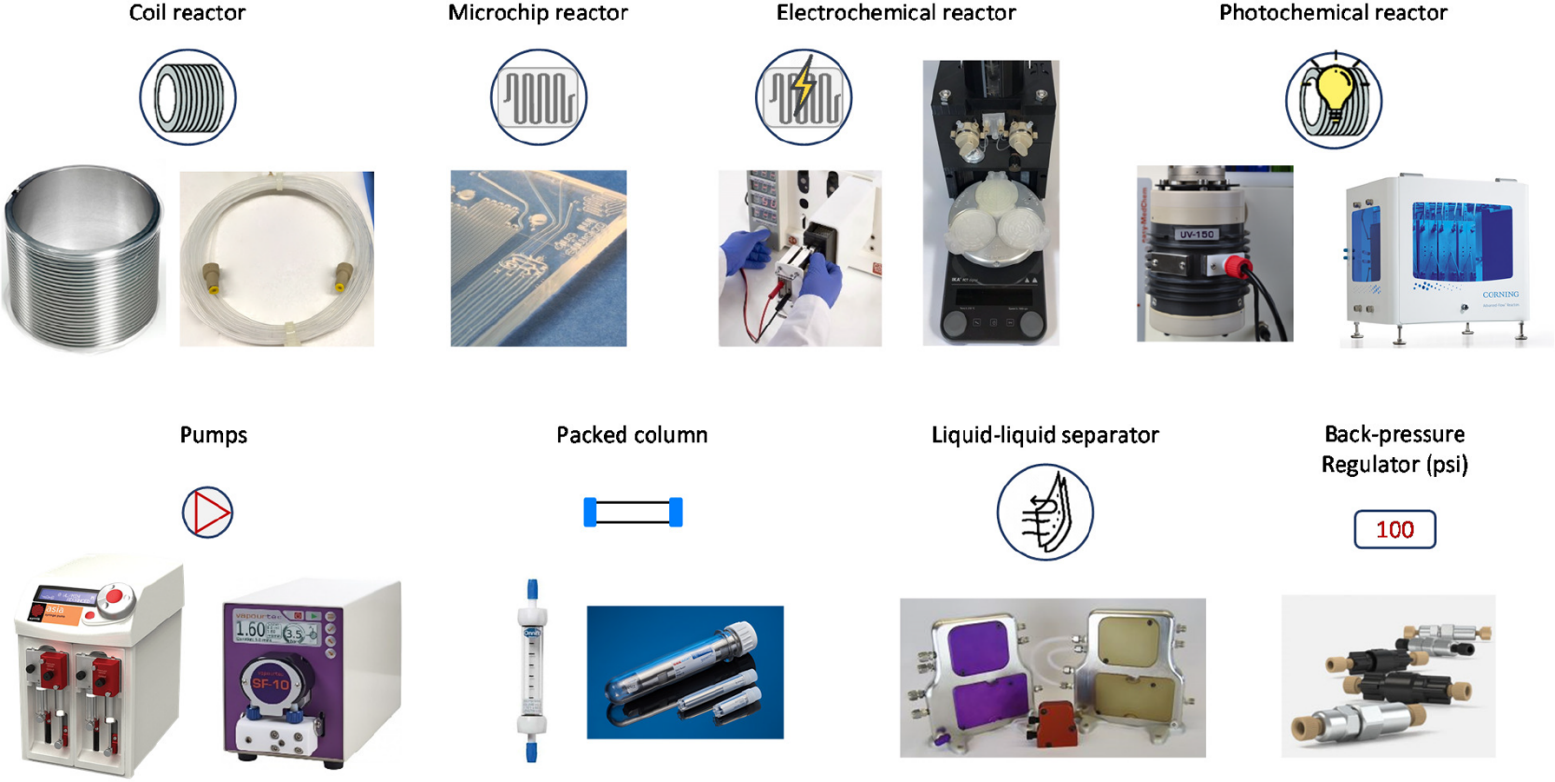
Examples of flow chemistry equipment.^39^

A typical flow apparatus consists of modular components that can be arranged interchangeably and repetitively, resulting in diverse adaptable combinations and set-ups. The connections between different modules utilize tubing and nonwetted parts, namely nuts and ferules, used to connect the tubing to the respective unit. The dimension, geometry, and type of material of tubing have to be properly chosen according to the working system pressure, chemical compatibility, and needs. In general, for low and medium pressure (<30 bar) inert perfluorinated polymers are appropriate, while high-pressure processes require more robust materials as stainless steel. Depending on the flow rate, the system pressure, and the nature of the reaction solutions, different types of pumps can be used to accurately feed starting materials and reagents into the flow system, including HPLC, syringe, peristaltic, and rotary pumps (Figure 3). HPLC pumps can be used in low- and high-pressure setup with flow rates higher than 0.1 mL min^–1^, although with volatile solvents pumping trouble are likely to occur. For lower flow rates in low pressure regimen, syringe pumps may ensure a better control. These pumps consist of two independent syringes, of which one is delivering predefined amount of liquid into the system while the second is being filled at the same time. With both HPLC and syringe pumps, the pumping system is in direct contact with the liquid phase, and therefore damages or interruptions due to reagent incompatibility, fouling, or precipitation may occur. Should these problems occur then peristaltic pumps may be an alternative for pumping at high flow rate and low pressure (up to 10–15 bar) also well-suspended slurries. Rotary pumps are another option: they can operate at higher pressure than peristaltic pumps and are endowed with a greater chemical and mechanical resistance than HPLC devices.

Reagents can be delivered into the system straight from the pumps or preloaded into sample loops. In this case, sample loops are connected to the flow system by six-way injection valves and can be associated with autosampler for automated reagent delivery. By the pump action, streams of reactants are pumped through the reactor after mixing by T- or Y-shaped connectors or by well-designed micromixing units for highly reactive transformations (Figure 3). Reactions occur within chip, coil, and packed-bed reactors whose working temperatures are tightly varied by means of thermocouples, cryogenic units, microwave irradiation, and inductive heating techniques (Figure 3).^23^ Selection of reactor type and material depend on the nature of the reaction and reactants.

Chip-based reactors are made from silicon, glass, ceramics, or stainless steel and guarantee a better control over mass and heat transfer, albeit with a low production rate and potential clogging issues. Coil reactors are manufactured from fluoropolymer (polytetrafluoroethylene [PTFE], perfluoroalkoxy alkane [PFA], and fluorinated ethylene propylene [FEP]) or stainless steel with different outer and inner diameters. Both typologies can be realized with light transparent materials to perform photochemical reactions.^21^ More recently, tube-in-tube, photochemical, and electrochemical reactors are commercially available to perform photo- and electrochemistry and reactions with gases. Finally, packed-bed reactors are ideal for heterogeneous catalysts or solid-supported reagents. Glass, polymeric, and stainless-steel columns or cartridges can be packed with solid materials to conduct heterogeneous catalysis or crude purification by scavenger resins. A special valve, namely back pressure regulator (BPR), maintains constant the pressure of the system, allowing working under superheated conditions while addressing safety concerns that may occur under conventional batch methods. At this point, the reaction stream can be analyzed before collection or enter downstream operations, including liquid/liquid separation by means of membrane or gravity separators, chromatography (simulated moving-bed chromatography), solvent switching, in-line evaporation, crystallization, and distillation.^23,40,41^

### 2.2 Process Analytical Technology

**Figure 4:**
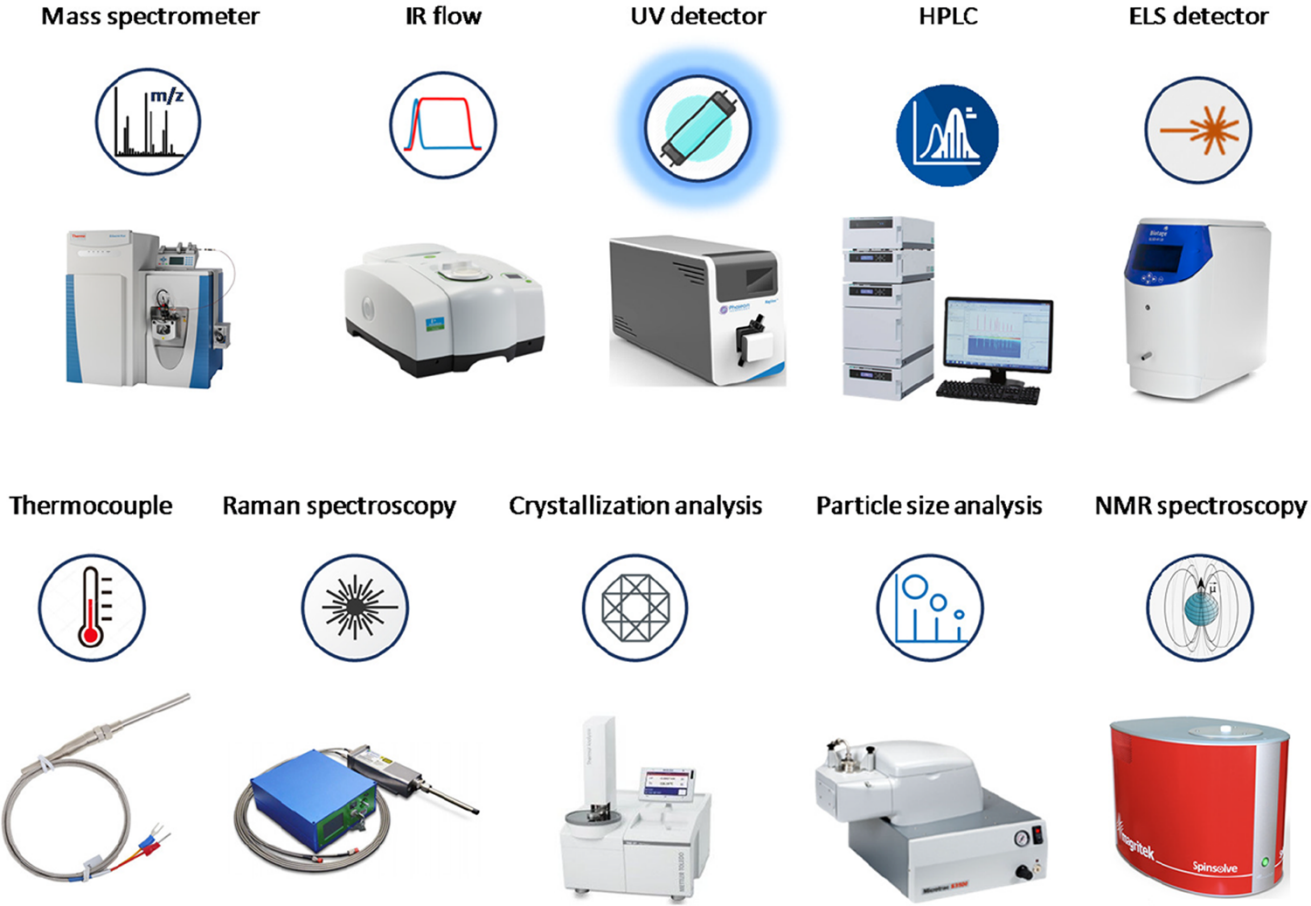
Representative examples of available PAT for continuous flow processes.^66^

The FDA has defined process analytical technology (PAT) as “a system for designing, analyzing, and controlling manufacturing processes through timely measurement of critical process parameters (CPP) which affect critical quality attributes (CQA)”.^42^ PAT includes a vast array of chemical, physical, and statistical analyses, as well as various analytical measurements, including thermocouple, infrared, Raman, and UV spectroscopy, mass spectrometry, chromatography, nuclear magnetic resonance, crystallization monitoring, and particles size analysis (Figure 4).^43−45^

Process control using PAT can be applied for real-time analysis, multivariate and simultaneous assessment of diverse quality parameters (quality-by-design, QbD), and for the careful control of process hazards.^46^ When integrated with flow technology, PAT is extremely useful for monitoring of telescoped synthesis, library building, and process optimization during scale-up development.^47−53^ With the assistance of software, PAT can operate with downstream devices for automated synthesis and with feedback systems to control reaction conditions and optimize in lieu of screening experiments.^54,55^

In-line analysis is realized via connecting the suitable analytical device in series so that the reaction mixture is analyzed after leaving the reactor. Alternatively, the analysis can be preceded by sampling using switching valves and fluid diverting devices for online and off-line analysis.^54−57^ Sample dilutors, solvent switching devices, and solvent removal apparatus can be located between sampling and analysis.

Numerous analytical techniques, tools, and sensors are currently available and applicable for flow devices depending on specific requirements of the process under investigation (Figure 4).^58^ High performance liquid chromatography (HPLC) and gas chromatography (GC) are easy-to-use techniques thanks to the readily availability of off-line versions in most of laboratories, low cost, high versatility in terms of detectable array of chemicals, and low implementation time.^59,60^ Optical spectroscopy represents by far the PAT of choice for fluidic processes^61^ and has been applied to a wide range of chemical transformations. Optical sensors and devices can be inserted directly inside or alongside flow reactors, thus avoiding material sampling. Depending on the required sensitivity, selectivity, and sample stability, different optical spectroscopic techniques can be coupled with flow machines, including UV–visible,^62^ fluorescence,^63^ Raman,^64^ and infrared spectroscopy (Figure 4).^65^ The integration of flow chemistry with high-resolution PAT detectors, as benchtop mass spectrometry (MS) and nuclear magnetic resonance (NMR), has made possible the real-time quantification and identification of reaction components in high-throughput fashion.^48−52^ However, compared to the most common optical spectroscopy and chromatographic techniques, both MS and NMR are more expensive and suffer from matrix effect, thus requiring, in most of the cases, a sampling step prior analysis to avoid undesirable interferences.

### 2.3 Computational Tools and Software

An autonomous flow-based machine works with the assistance of computational tools and software. Statistical programs such as design of experiments (DoE), evolutionary, self-optimizing, or machine learning algorithms and cloud-based systems have been demonstrated to be efficacious to monitor, manage, and fine-tune operating flow systems for both medicinal chemistry and organic synthesis applications.^29,30,67−75^ Beyond the simple management of reagent selection and compound collection for library building, machine-assisted flow devices can be also applied for predictive and decision-making actions in closed-loop mode for both medicinal chemistry learning and process optimization.^12,31^ Although this area is still in a nascent state, recent advances have propelled different manufactures of flow equipment, research groups, and specialized companies into the development of specific software and programming languages for automated drug discovery platforms.^76^ Open-source software and computer-aided approaches for automating flow systems are also rapidly growing and include suites as LabVIEW,^77^ MatLab,^78^ LeyLab,^29^ OpenFlowChem,^60^ ChemOS,^79^ and Chemputer.^80^

LabVIEW (Laboratory Virtual Instrumentation Engineering Workbench) is a graphical programming language developed in 1986 by National Instruments for automation control and data acquisition.^81^ The graphical representation is based on intuitive process diagrams and composed of three main components: a front panel, which is an input-containing module, a block diagram, which allows editing of codes that will be visualized graphically, and a connector panel which serves as the interface of connection. This apparently simple network allows the integration and automation of systems, drivers, and benchtop applications also in remote fashion through multiple controls. This programming language, which has been continuously implemented over the years up to the current version 19.0, includes a multitude of data analysis and process control functions, different code frameworks (as COM,.NET and shared DLL), as well as different communication protocols (e.g., RS232, GPIB, and TCP/IP). LabVIEW has gained a prominent role for instrument control, system integration, robotics, automation, and database.

MatLab is an open-access software distributed by Mathworks Inc. that enables customizable and advanced data analysis.^78,82^ Numerous works^54,55,83−89^ have demonstrated the profitable integration of MatLab code and LabVIEW programming language for implementing autonomous and fully integrated flow platforms for reaction screening and optimization and for medicinal chemistry purposes, as discussed later in this Perspective.

**Figure 5:**
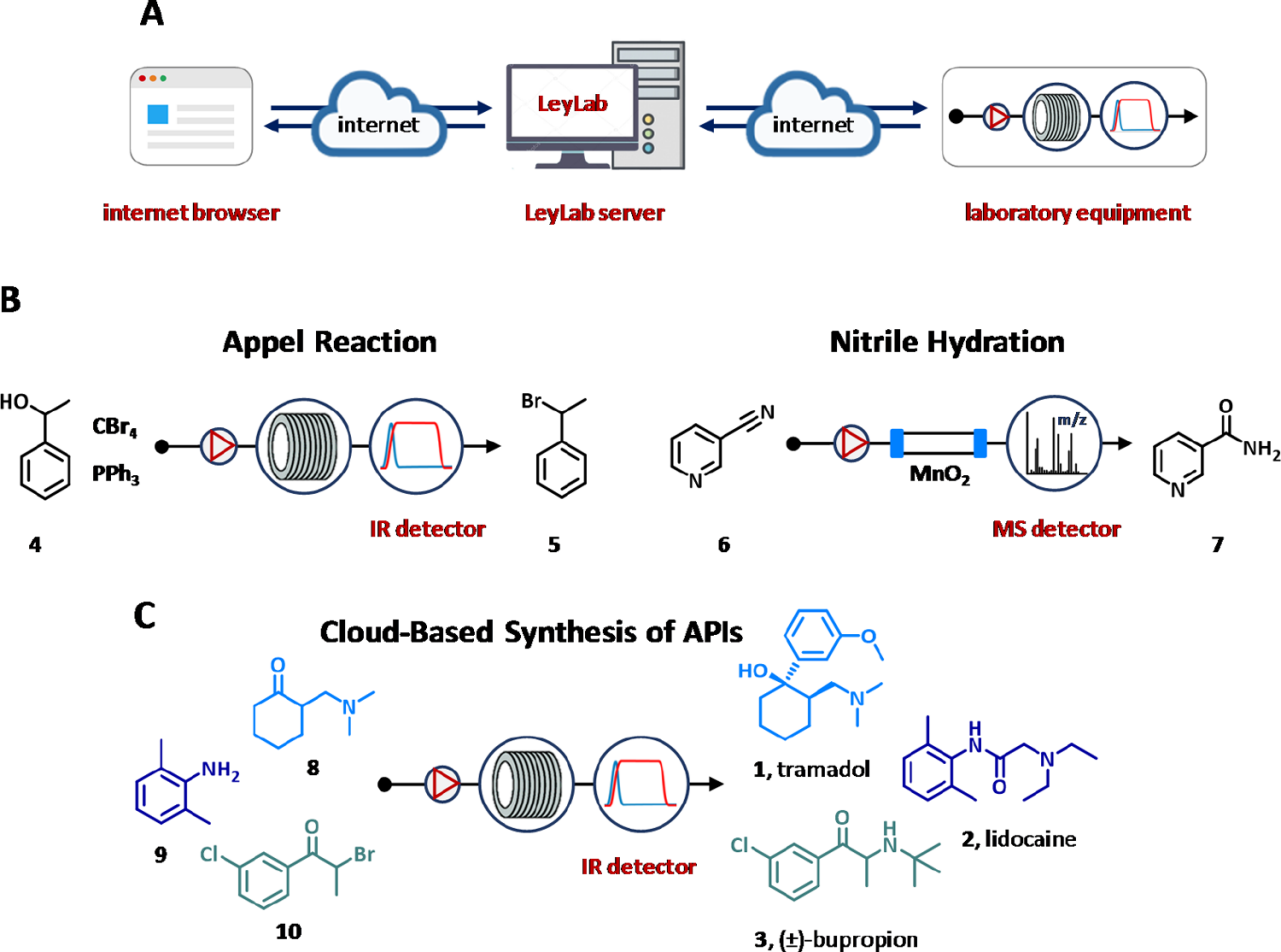
Schematic representation (A) and case-studies (B,C) of the LeyLab.

In 2016, Ley’s group developed LeyLab, a software based on the Internet of Things (IoT) concept for the remote control and monitoring of automated flow platforms working with both user–server and server–equipment communication by TCP/IP protocol (Figure 5A).^29^ The software featured a graphical interface accessed via internet browser, a database for storing all the information and data related to the experiments and equipment, a communication module that comprises different codes, protocols, and commands, and a command module containing all the code definitions and commands for individual equipment. The software performance has been successfully adopted for the multidimensional optimization of the Appel reaction and nitrile hydration reaction using in-line IR and MS analysis, respectively (Figure 5B).

In addition, LeyLab allows locating and controlling equipment in different places from the server as demonstrated for the synthesis of three diverse APIs (Figure 5C).^90^ Self-optimizing reactions, which included the Grignard addition for the synthesis of (±)-tramadol (**1**), the amine cyclization and alkylation on the way to lidocaine (**2**), and the bromination/amine alkylation step for preparing (±)-bupropion (**3**), were monitored and controlled in Los Angeles (CA, USA) using equipment in Cambridge (UK) via servers located in Japan (Figure 5C).

**Figure 6:**
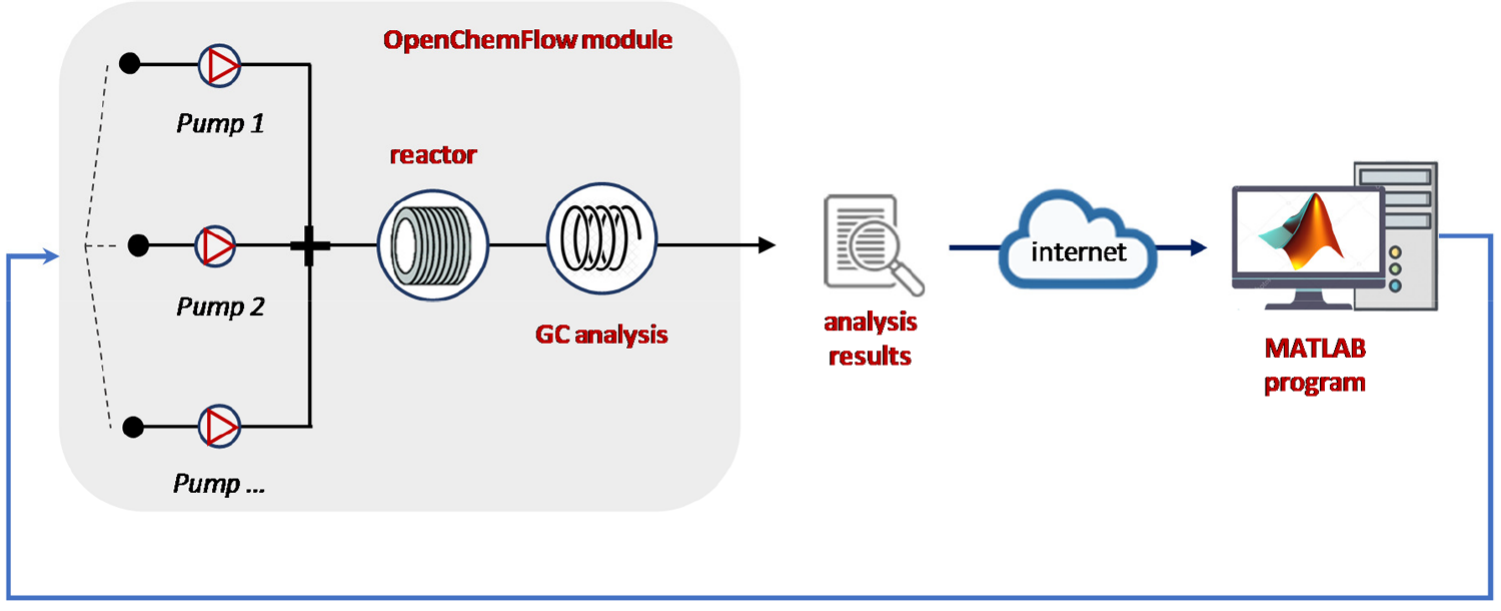
Schematic representation of the OpenFlowChem platform.

More recently, OpenFlowChem,^60^ an open-source platform for process automation, control, and monitoring was created with the aim to simplify the combination between different software (Figure 6). On the basis of LabVIEW and cloud-based data transfer with MatLab for optimization via the SNOBFIT algorithm, this flexible platform requires less programming efforts to modify the initially configured setup. The OpenFlowChem platform is composed by a device monitor able to handle the connected equipment, a system module that provides the integration among the instruments, and an optional external safety device.

ChemOS is a versatile, flexible, and modular software package recently developed to combine and orchestrate autonomous robotics platforms with AI algorithms.^79^ The platform also supports remote control of equipment and performing experiment in parallel across laboratories located in different countries. ChemOS consists of six modules that include: (i) interaction with researchers, (ii) databases handling and managing, (iii) robotics, (iv) characterization, (v) learning procedures, and (vi) analysis. The core of this system is the learning module, which is able to autonomously and continuously propose new sets of parameters for novel experiments on the basis of previous outcomes.

**Figure 7:**
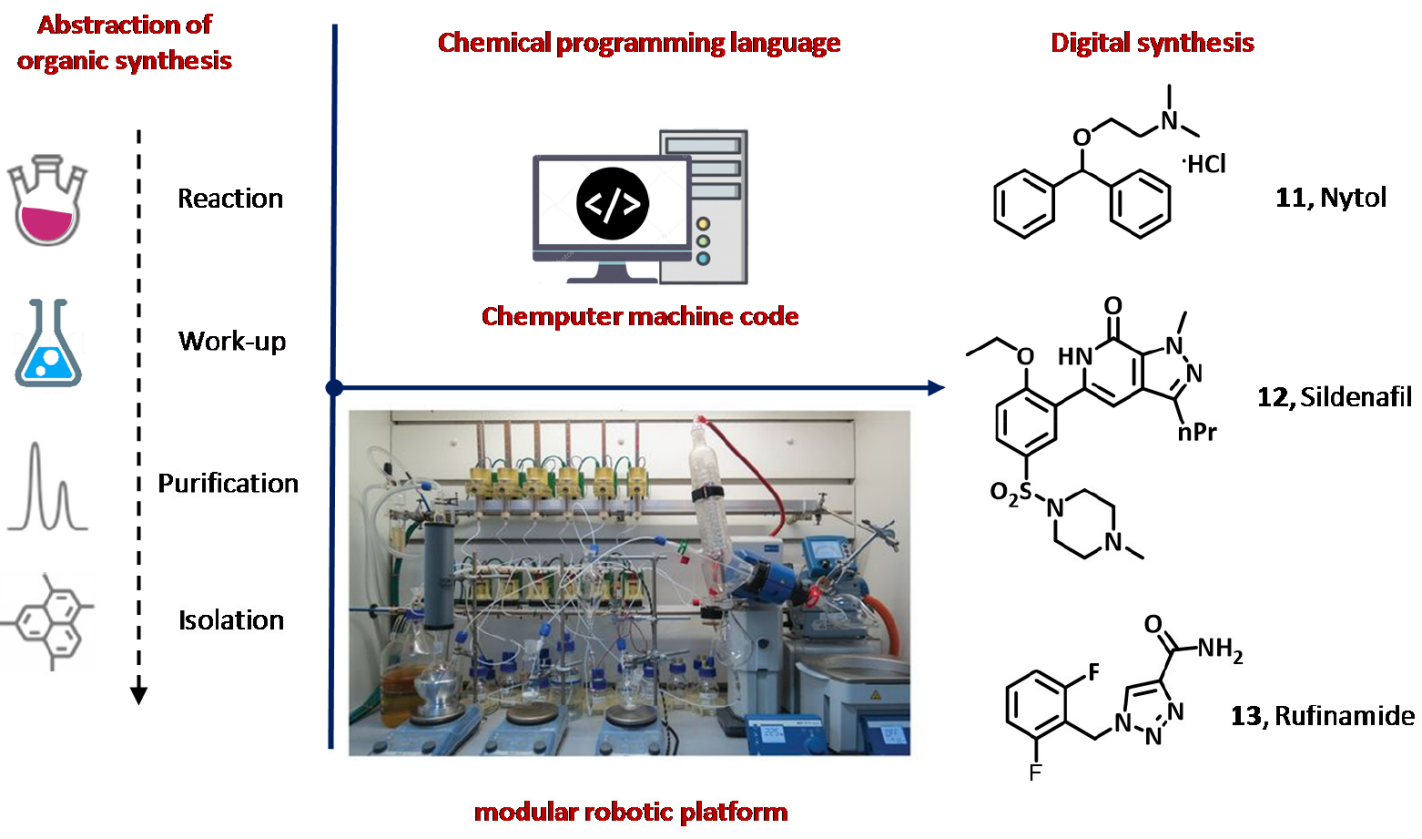
Schematic representation of Chemputer used for the synthesis of diphenhydramine hydrochloride (Nytol, **11**), sildenafil (Viagra, **12**), and rufinamide (Banzel, **13**). Reproduced with permission from ref (80). Copyright 2019, American Association for the Advancement of Science.

Finally, Chemputer is a software suite recently developed by Cronin’s group.^80^ Although to date Chemputer has been applied for round-bottom flask chemistry, the software is capable of controlling whole hardware modules and to combine individual unit operations required to accomplish the laboratory-scale automated and multistep synthesis of the desired chemical compounds. Programmable machine operations and chemical processes include heating/cooling systems, the control of stirring and pump operations for reagent additions and mixing, system cleaning and priming, reaction quenching, filtration and biphasic liquid–liquid extraction, and vacuum operation for rotary evaporation (Figure 7). Chemputer was shown to control complex multistep synthetic sequences using procedures collected from the Reaxys database without any human intervention as proved for the synthesis of diphenhydramine hydrochloride (Nytol, **11**), sildenafil (Viagra, **12**), and rufinamide (Banzel, **13**).

### 2.4 Biological Assays

The integration of flow platforms with biological assays offers an opportunity to solve compartmentalization and spatiotemporal boundaries, reducing idle times in medicinal chemistry cycles. Moreover, in-line flow testing requires small volumes (nL or pL) of the test solution and has shown improving data reproducibility.^91^ Several reports have disclosed the development of specific bioassays compatible with flow systems and the creation of microfluidic lab-on-chip devices for chemical biology investigations.^92−94^

**Figure 8:**
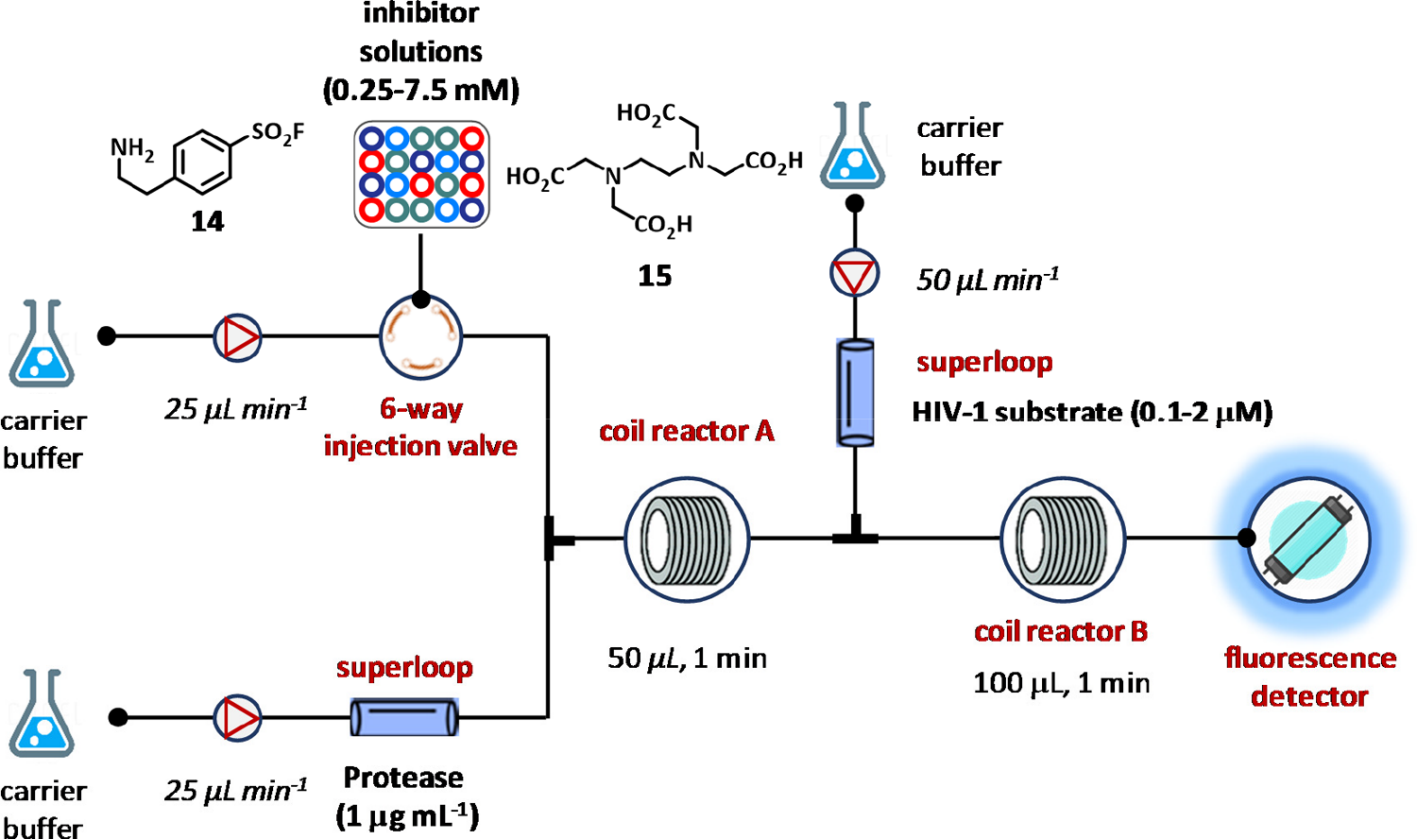
Representation of an homogeneous continuous flow assay using fluorescence resonance energy transfer (FRET). The enzyme solution and the carrier buffer, delivered at 25 μL min^–1^ for each syringe pump, were mixed in coil reactor A. A superloop was placed between syringe pump 1 and coil reactor A, while an autoinjector was placed between syringe pump 2 and coil reactor A. The enzyme/inhibitor mixture from coil reactor A was mixed with HIV protease substrate 1 delivered by pump 3, which was connected to an inverted Y-piece to reduce the flow rate at 50 μL min^–1^. Excitation and emission wavelengths for the fluorescence detector were 340 and 490 nm, respectively.

One of the first studies dates back to 2003, when Hirata and collaborators described a homogeneous continuous-flow assay consisting of fluorescence resonance energy transfer (FRET) to measure the hydrolysis of human immunodeficiency virus (HIV) protease substrate 1 in the presence of two inhibitors, namely 4-(2-aminoethyl)benzenesulfonyl fluoride hydrochloride (AEBSF, **14**) and ethylenediaminetetraacetic acid (EDTA, **15**) (Figure 8).^95^ The HIV protease substrate 1 was covalently bound with two chromospheres, namely EDANS (donor) and DABCYL (acceptor). The system consisted of pumps, two Superloop and PTFE coil reactors, an automated injector with a six-port injection valves, and a fluorescence detector within the appropriate flow cell (Figure 8). Under optimized conditions, the enzyme solution (1 μg mL^–1^) and the carrier buffer (0.1 M phosphate buffer, 0.1 M sodium chloride and 0.05% (v/v) Tween 20 at pH 7.5) were pumped into the first reactor coil at 25 μL min^–1^. A Superloop was fitted between the first syringe and the coil reactor to deliver the enzyme solution into the system, while the automated injector with a six-port injection valve was placed between the second syringe and the first reaction coil for injecting the inhibitor solutions at concentrations ranging from 0.25 to 7.5 mM. The outcome was then combined with the stream of the HIV protease substrate-1 (0.1–2 μM) delivered at 50 μL min^–1^ to enter a second coil reactor. Finally, the mixture eluted to a fluorescence detector to assess enzyme inhibition and generate dose–response activity (Figure 8). The microfluidic bioassay was later implemented with the insertion of online coupling with size-exclusion liquid chromatography (LC) technique useful to separate protease inhibitors aprotinin and AEBSF (**14**) from inactive compounds.^96^

**Figure 9:**
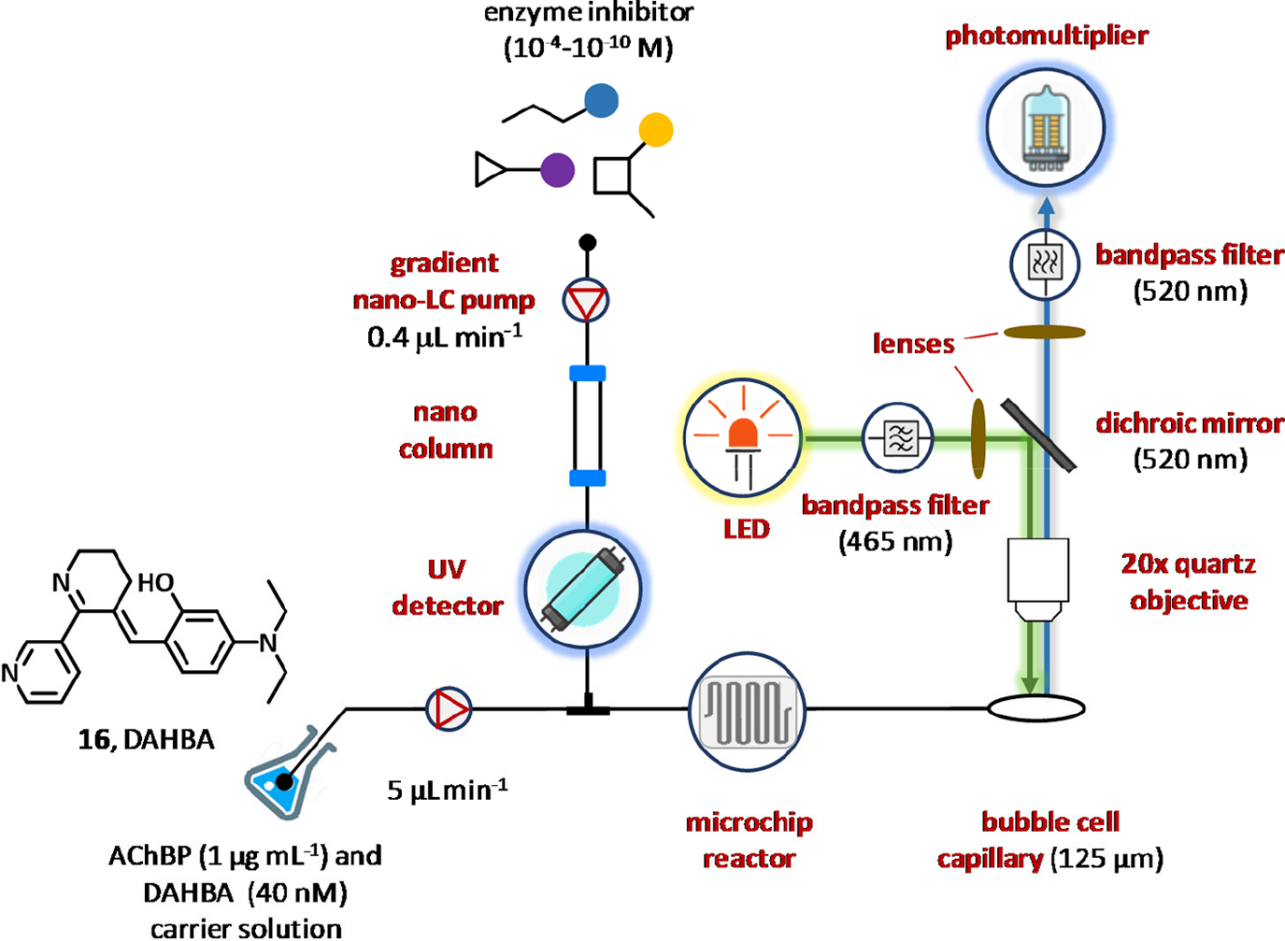
Development of a microfluidic confocal fluorescence detection assay for the identification of acetylcholine binding protein inhibitors. The bioassay carrier solution containing AChBP and DAHBA (**16**) was delivered at 5 μL min^–1^ and mixed in a microfluidic chip (4 μL) with the nano-LC effluent containing the potential protein ligand pumped at 0.4 μL min^–1^. When DAHBA (**16**) is displaced by eluting protein ligands, a bubble cell capillary in the reaction chamber of the miniaturized chip detects fluorescence variation by means of a photomultiplier tube.

In 2010, Heus and colleagues reported a new method for minimizing sample and reagent consumption by coupling online nano-LC to a light emitting diode (LED) and a capillary confocal fluorescence detector.^97^ The hyphenated technique was successfully applied for the identification of acetylcholine binding protein inhibitors (Figure 9). In particular, the bioassay solution containing acetylcholine binding protein (AChBP, 1 μg mL^–1^) and (*E*)-3-(3-(4-diethylamino-2-hydroxybenzylidene)-3,4,5,6-tetrahydropyridin-2-yl)pyridine (DAHBA, **16**, 40 nM) as the fluorescent tracer ligand, was delivered by a syringe pump at a flow rate of 5 μL min^–1^ and mixed in a miniaturized chip (4 μL) with the nano-LC effluent pumped at 0.4 μL min^–1^. Inside the biochemical reaction chamber, DAHBA is eventually displaced from the acetylcholine binding protein by the potential ligand eluting from the nanocolumn, thus resulting in a decrease of fluorescence. The detection unit consisted of a high intensity LED lamp, a series of excitation and emission filters, a confocal lens, a dichroic mirror, a photomultiplier tube, and a bubble cell capillary. Light emitted by LED lamp was filtered through a 465 nm single bandpass filter, collimated by a lens, and reflected of 90° into the bubble cell by a dichroic mirror (520 nm). The emitted light then passed the same dichroic mirror, focusing lens, and a 520 nm single bandpass filter and was finally detected by the photomultiplier tube. The detection system was coupled with a gradient reversed-phase nanoliquid chromatography operating under flow injection analysis modality and in a concentration–response fashion. Overall, the combination of such equipment allowed to determine the IC_50_ values of nine inhibitors using only 10 nL for each compound that corresponded to ∼100 pmol.^97^

**Figure 10:**
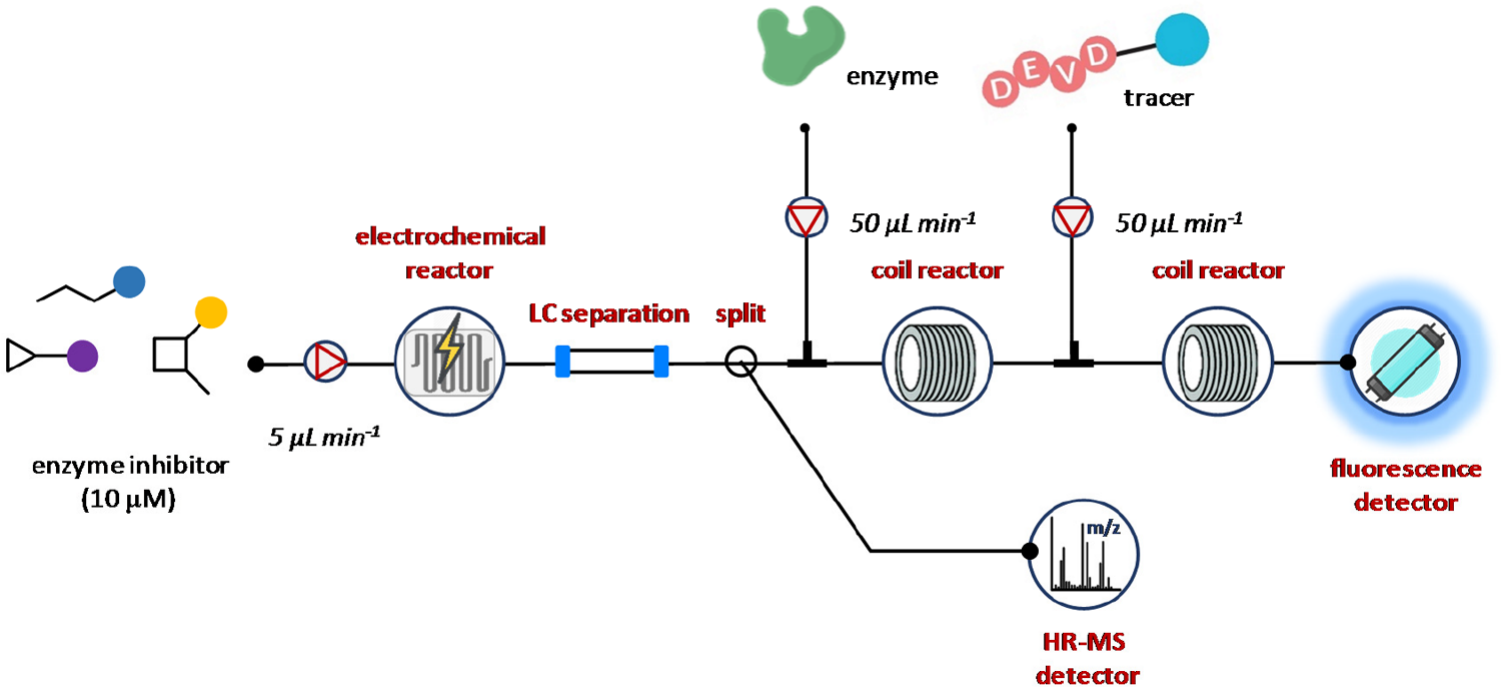
Integration of electrochemical reaction cell with a continuous flow bioaffinity assay and LC-HRMS analysis. Different potentials (0, 0.4, 0.8, 1.2, and 1.5 V) and operative pH (3.5, 5.0, 7.0, and 10.0) were evaluated for the electrochemical conversion of each substrate.

Researchers at Amsterdam University provided an excellent proof-of-concept for the integration of hyphenated electrochemical reaction cell with a continuous flow bioaffinity assay and LC-HRMS to determine and characterize electrochemical conversion products as p38α mitogen-activated protein kinase inhibitors (Figure 10).^98^ The system consists of four modules: an electrochemical reaction cell, a LC system, a continuous flow bioaffinity assay unit equipped with a fluorescence detector, and a mass spectrometer. Standard solutions of kinase inhibitors dissolved in the appropriate buffer (25% MeCN and 75% of 1 mM aqueous buffer) at 10 μM final concentration were delivered at 5 μL min^–1^ by a syringe pump. After the online electrochemical conversion of the inhibitor, a gradient LC column was fitted for the separation of the products formed. A postcolumn valve was used to split the eluate through the p38α bioaffinity assay and the MS system. Thus, part of the eluate (13 μL min^–1^) was mixed with p38 kinase α, delivered at 50 μL min^–1^, and directed into the reaction coil for enzyme binding. At the same time, a second aliquot deriving from the electrochemical conversion (100 μL min^–1^) was analyzed in-line using a Shimadzu ion trap time-of-flight hybrid mass spectrometer (LC-IT-TOFMS) to gain structural information on binders. Finally, after the addition of tracer molecule, the detection of enzyme–tracer complex by fluorescence allowed the rapid characterization of novel p38a kinase inhibitors (Figure 10).

**Figure 11:**
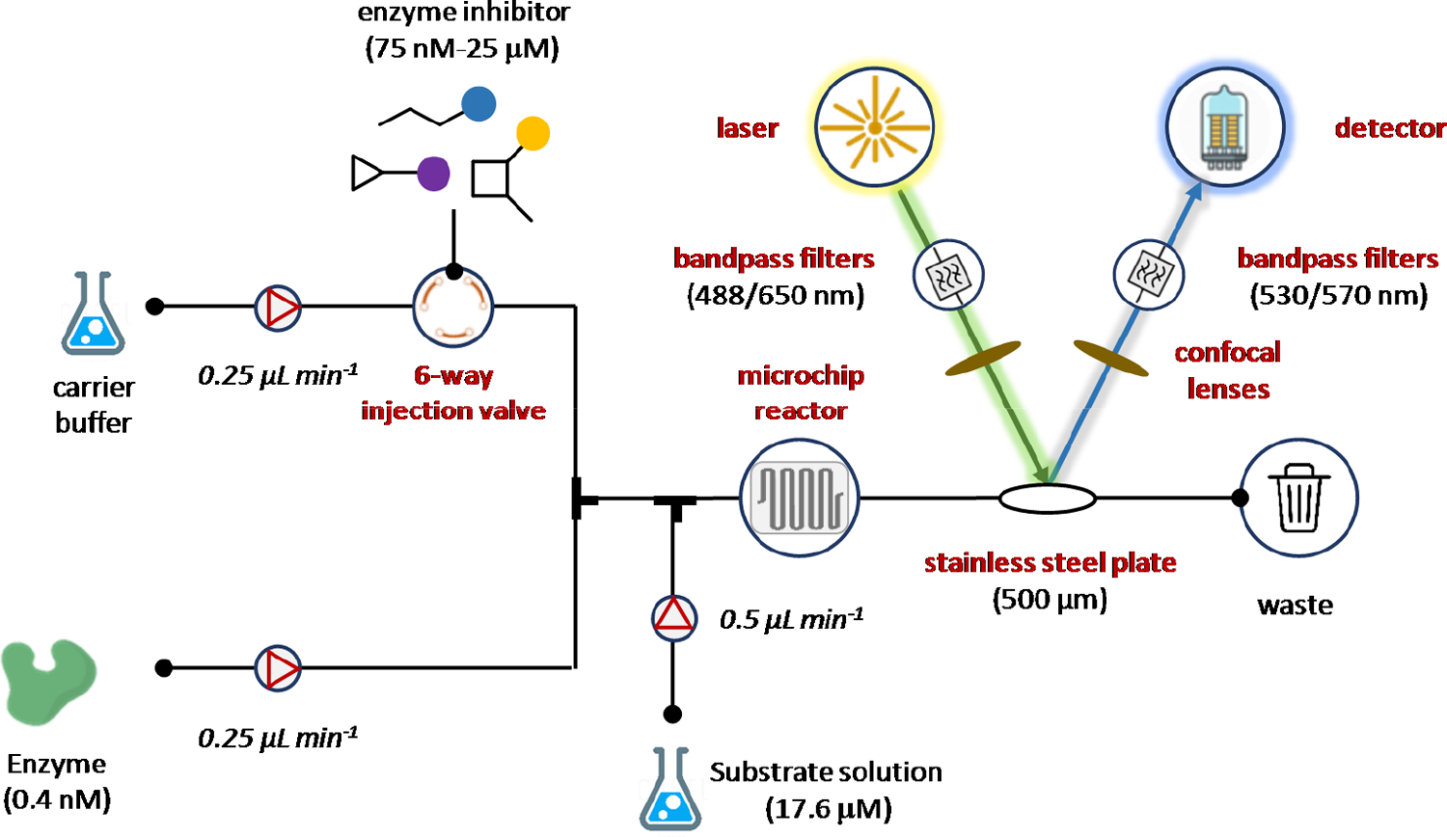
Microfluidic continuous-flow injection titration assay (CFITA) for monitoring inhibition of thrombin peptidase activity. Enzyme inhibitor solutions were prepared and tested by dissolving the screened compounds at concentration ranging from 75 nM to 25 μM using a buffer (50 mM HEPES pH 8.0 containing 1 mM calcium chloride, 142 mM sodium chloride, 0.05% CHAPS, and 0.1% trehalose) containing 500 nM of Cy5 dye as the internal standard.

More recently, Patel and co-workers developed a microfluidic continuous-flow injection titration assay (CFITA) for monitoring inhibition of thrombin peptidase activity (Figure 11).^99^ The CFITA assay equipment was based on a four-channel pumping system for delivering enzyme, substrate, buffer, and the tested compounds. In particular, thrombin (0.4 nM) and its substrate (17.6 μM) were both pumped at 250 nL min^–1^ using specific milliGAT LF pumps for high precision fluid handling. Check valves and adapters were connected following the pumps in order to prevent back-flow and for reducing tubing diameter. A six-way injection loop was filled with the tested inhibitors diluted with the assay buffer containing Cy5 dye (500 nM) as the internal standard. Inhibitor titration was therefore started by switching the valve from “load” to “injection” position and, at the same time, an additional pump delivered the buffer into the inhibitor channel at 500 nL min^–1^ total flow rate to generate the gradient. A digital flowmeter guaranteed the monitoring of the total system flow rate (1 μL min^–1^). The bioassay occurred into a stainless steel plate, while the optical equipment was composed by bandpass filter confocal lenses endowed with two excitation (488/650 nm) and two emission (530/670 nm) dichroic filter wavelengths, a laser module that induced the simultaneous excitation, and a set of aspheric confocal lenses for baseline correction. As a result, step gradient titration curves for bioassay data and gradient data were generated for each tested compound. This flow biochemical assay has been recently integrated into the Cyclofluidic closed-loop drug discovery flow platform.^100^

Finally, numerous examples of droplet microfluidic assays for synthetic biology applications, including DNA assembly, transformation/transfection, culturing, cell sorting, phenotypic assays, artificial cells and genetic circuits have been reported and recently reviewed by Gach et al.^101^

## 3 Automated Flow Synthesis and Off-Line Compound Testing

**Figure 12:**
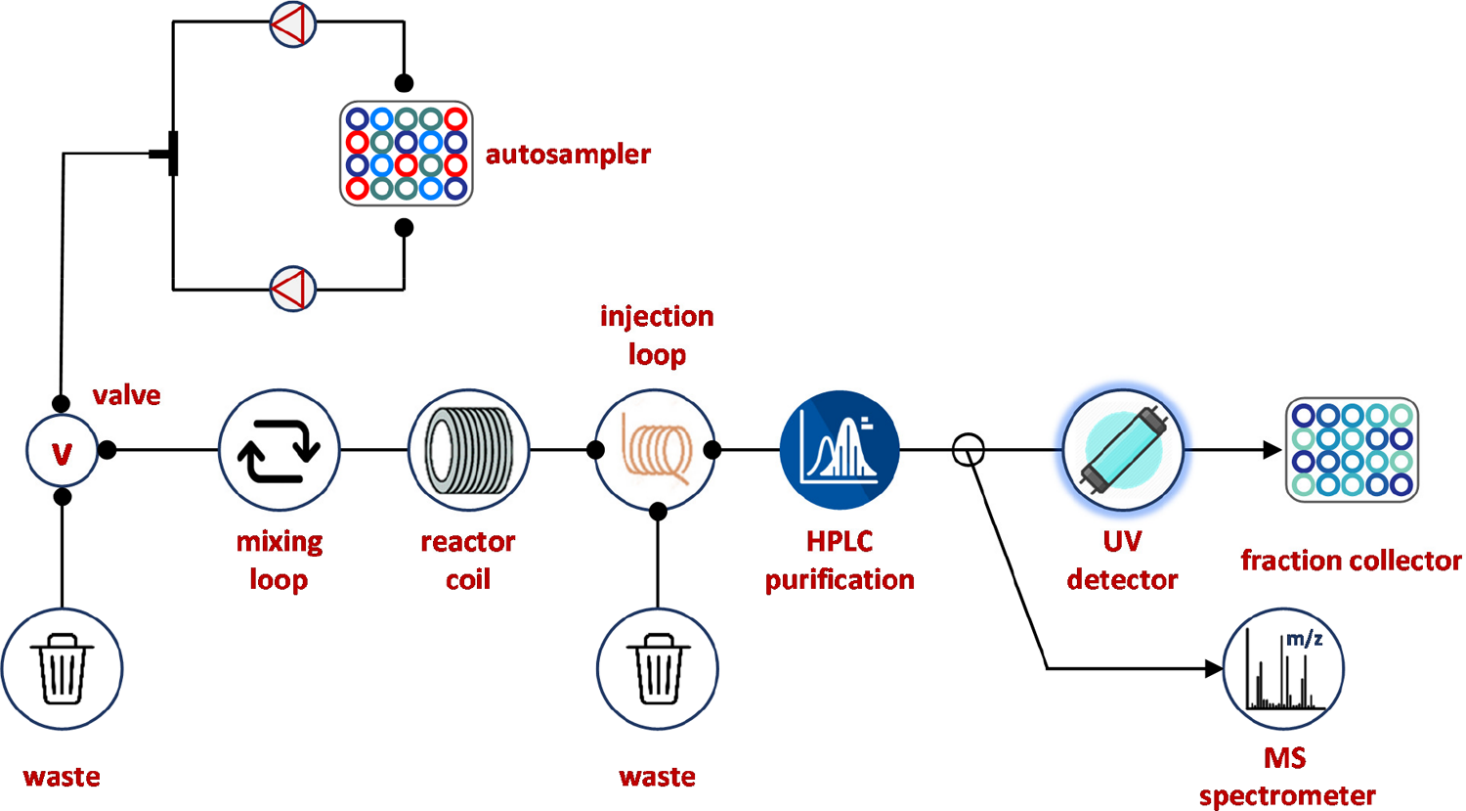
Schematic diagram of the SWIFT system from AbbVie.

In the past few years, a number of examples have been reported showing the profitable use of automated flow synthesis of compound collections readily available for biological testing.^102−116^ Among these, it is worth mentioning the system realized by Djuric and co-workers at AbbVie in 2011 is probably one of the first fully integrated systems for the generation of diverse classes of compounds under automated flow modality (Figure 12).^113^ The system, namely SWIFT (Synthesis With Integrated Flow Technology), was based on an Accendo Conjure flow reactor integrated with autosampler and preparative HPLC/MS device (Figure 12) to conduct different chemical transformations, including amide bond and urea formation, reductive amination reactions, Huisgen cycloaddition for triazole synthesis, nucleophilic displacement, and sulfonylation reactions and to obtain highly pure products. In particular, the setup enabled the synthesis of diverse chemical libraries composed of 10–48 members at 10–20 mg scale with yields ranging from 23% to 63% and an average throughput of six compounds per hour. In 2014, SWIFT was further implemented with a Mitsubishi robot (Figure 12) that collected samples throughout the process to the next stage, including synthesis, purification, sample dispending for purity assessment, evaporation, and sample preparation for screening.^110^

**Figure 13:**
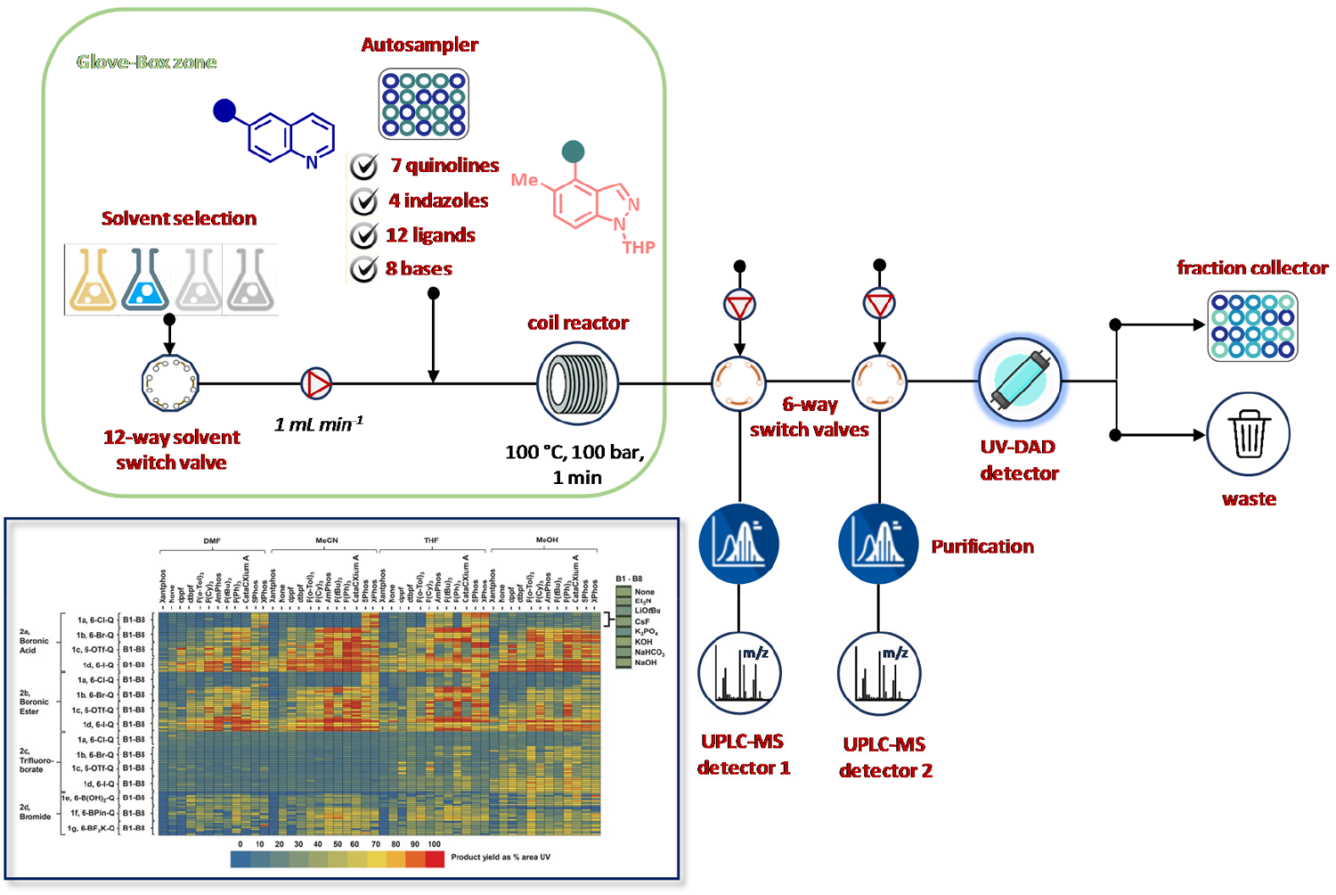
Automated flow platforms for Suzuki–Miyaura reaction screening, optimization, and validation. The system was composed by a 192 well plate autosampler connected with a HPLC system for reaction segment injection on a predefined sequence, a pump that generates the flow stream, a reactor coil, and a 12-port valve for solvent selection. The reaction outcome is directed by the six-port switching valve into the UPLC-MS instrument for reaction analysis. The excess sample is directed through a diode array detector (DAD) for product collection or waste. Reproduced with permission from ref (102). Copyright 2018 American Association for the Advancement of Science.

More recently, Perera and co-workers described how to combine strategies for reaction optimization with compounds synthesis and broaden the reaction chemical space in a medicinal chemistry setting.^102^ The automated flow-based platform was associated with an ultrafast experimental screening of Suzuki–Miyaura reaction with the synthesis of cross-coupling products (Figure 13). This single modular unit was validated by the screening of different reaction conditions and components including solvents, catalysts, and bases, thus generating information for about 6k reactions in less than four days. Optimal conditions were then applied for preparing Suzuki–Miyaura adducts at a milligram scale in good to excellent yields (≥85%) and high purity.^102^

The following case studies have been selected to showcase the development of high-throughput flow strategies for the synthesis and characterization of chemical libraries using different levels of automation and off-line biological evaluations. We therefore refer the interest of readers to previous reviews for the synthesis by automation of putative active compounds.^117−119^

**Figure 14:**
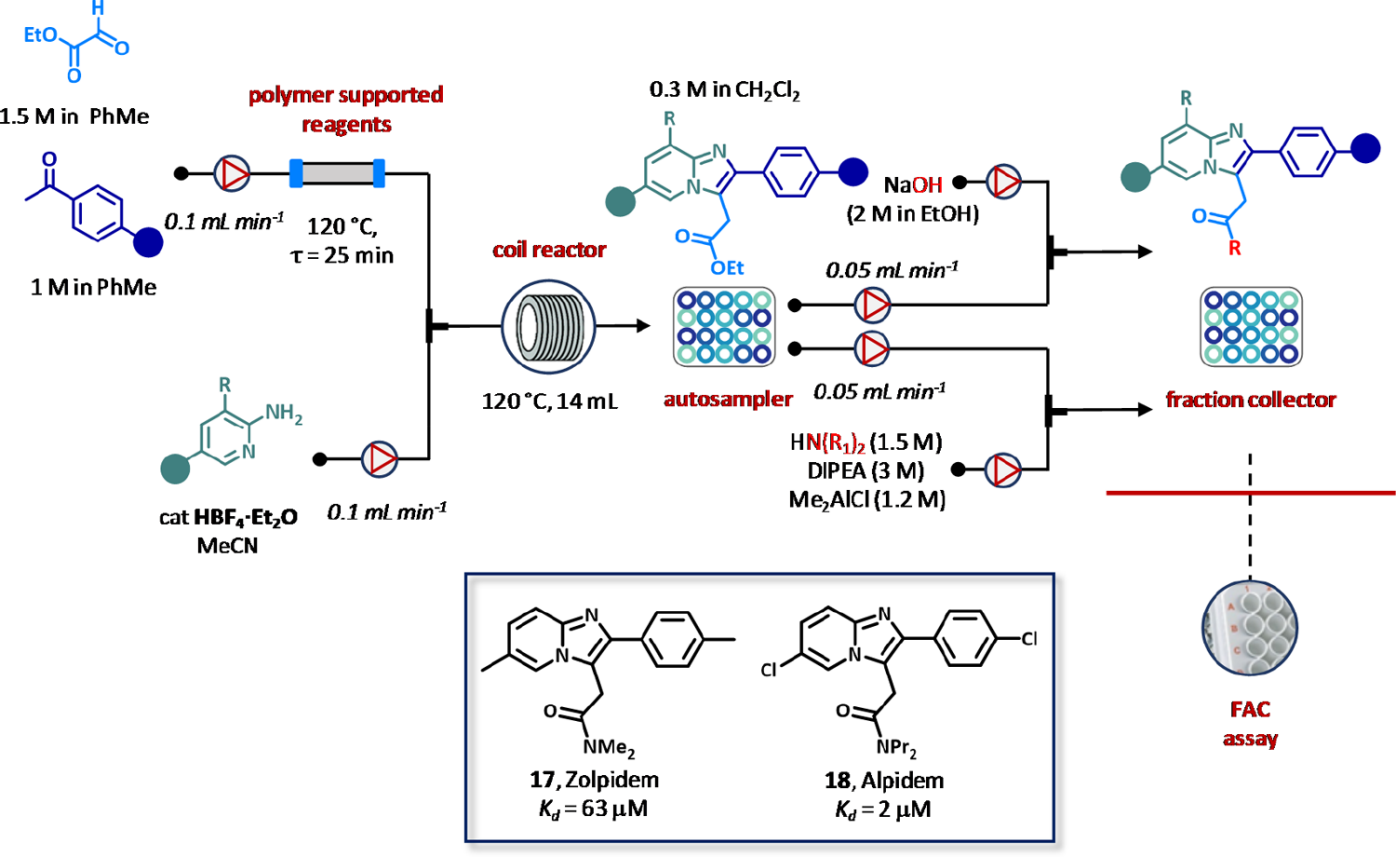
Automated flow synthesis and purification of imidazo[1,2-*a*]-pyridine tested by frontal affinity chromatography (FAC) assay. Products were prepared by acid-catalyzed condensation between ethyl glyoxylate and acetophenone analogues, followed by reaction with aminopyridines. Ketoimine intermediates thus formed were submitted to thermal cyclization and finally diversified by amidation or hydrolysis of the ester moiety. For each compound, three concentrations (7.81, 31.25, and 62.5 μM) were prepared by diluting the corresponding stock solutions (125 μM in PBS) and injected in triplicate into the HPLC. Determination of binding constants (*K*_d_) was performed at 254 and 262 nm in the presence of 20 nmol of HSA immobilized on the column.

In 2013, the Ley group described an automated three-step flow synthesis of imidazo[1,2-*a*]-pyridine derivatives, including GABA-A agonists zolpidem (**17**) and alpidem (**18**), tested by frontal affinity chromatography (FAC) for albumin binding evaluations (Figure 14).^120^ The procedure included the use of tubular and coil reactors, supported reagents and scavengers, autosamplers for reagent dosing, and fraction collectors. The synthesis started with the reaction of ethyl glyoxylate (1.5 M in PhMe) and acetophenone analogues (1 M in PhMe) induced by polymer-based sulfonic acid resin at 120 °C for 25 min. The excess of glyoxylate was easily removed using a scavenging column packed with supported benzyl amine. Pure unsaturated ketone intermediates were dosed at 0.3 M concentration by an autosampler and reacted with a slight excess of diverse aminopyridines on a tubular reactor filled with a MgSO_4_ heated at 50 °C (Figure 14). Ketimines thus formed readily underwent 5-exo cyclization at 120 °C within a coil reactor and the crude mixtures were successively purified by means of an acid resin. Library diversity was further improved by the conversion of the ester moiety into amide and carboxylic acid group. As a result, 22 imidazo[1,2-*a*]-pyridine analogues were prepared in four days in 10–70% yield. After manual or automated dilution, the synthesized compounds were flowed through a column containing immobilize human serum albumin (HSA) for FAC binding activity (Figure 14). Besides the high degree of correlation between the determined *K*_D_ values and previously reported data for zolpidem (**17**) and alpidem (**18**), the approach may be of utility to predict drug–serum protein interaction.

**Figure 15:**
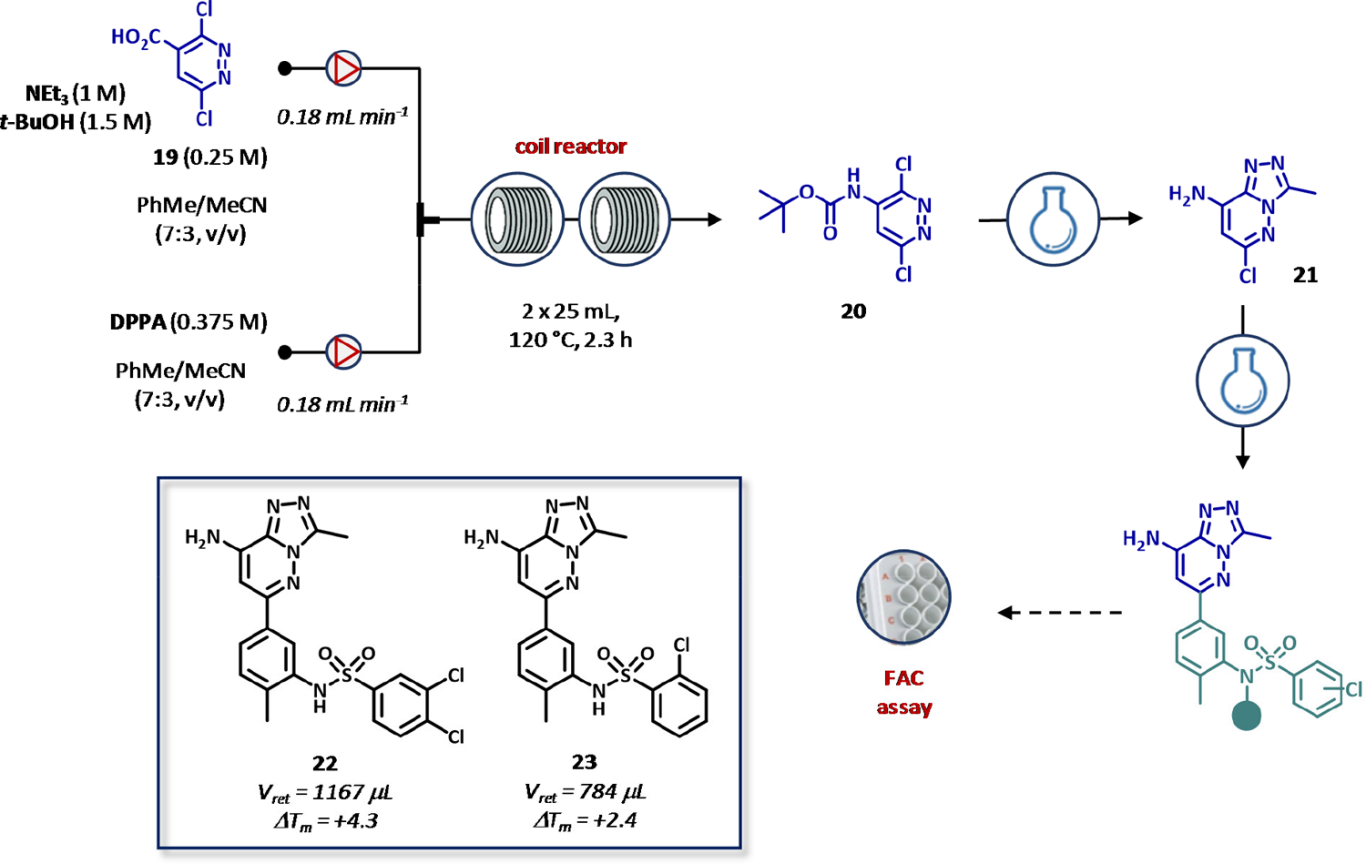
Automated flow synthesis and testing of BRD9 bromodomain inhibitors by frontal affinity chromatography (FAC) assay. Desired products were generated by in flow Curtius rearrangement of the in situ generated acyl azide intermediate, followed by *tert*-butyloxycarbonyl (BOC) deprotection, triazole ring formation, and Suzuki cross-coupling reaction under conventional batch conditions. Detection was performed at 220 and 254 nm. The amount of active loaded protein and the affinity constants were calculated by injecting in duplicate a solution of bromosporine in DMSO at concentrations of 15, 7.5, 3.75, and 1.875 μM, both in PBS and in 100 mM ammonium acetate buffer starting from a 50 mM stock solution.

The same tactic was adopted for the preparation of novel BRD9 bromodomain inhibitors.^121^ In particular, the in-flow Curtius rearrangement of 3,6-dichloropyridazine-4-carboxylic acid (**19**, 0.25 M) in the presence of Et_3_N (1 M), *t*BuOH (1.5 M), and diphenylphosphoryl azide (DPPA, 0.375 M) into intermediate **20** was instrumental for the multigram preparation of the amino triazolepyrazine core (**21**) later employed as building block for the synthesis of structurally related analogues (Figure 15). A remote-controlled flow equipment was used to generate the acyl azide intermediate that was submitted to a thermal cyclization (120 °C) in two stainless-steel reactors (50 mL, τ = 2.3 h). An in-line pressure regulator ensured the control of gases produced during the reaction and a better heat exchange compared to the conventional batch procedures. Most importantly, the unstable acyl azide intermediate was readily reacted to autonomously synthesize the carbamate **20** with a productivity of 2 mmol h^–1^. Next steps including *tert*-butyloxycarbonyl (BOC) deprotection, triazole ring formation, and Suzuki cross-coupling reaction were performed in batch mode, yielding six BRD9 bromodomain inhibitors. The synthesized compounds were then tested in off-line modality using a FAC-MS apparatus, which was specifically designed and adapted for the BRD9 assay and consisted of a customized column containing the biotinylated BRD9 bromodomain immobilized on streptavidin coated beads. As a result, compound **22** was identified as the most potent analogue of the series showing a retention time (*V*_ret_) of 1167 μL in FAC-MS assay and a thermal shift (Δ*T*_m_) of +4.3 in thermal stabilization assay against BRD9. Furthermore, albeit less potent (*V*_ret_= 784 μL, Δ*T*_m_ = +2.4), compound **23** showed a better selectivity over BRD4 bromodomain (Figure 15).

**Figure 16:**
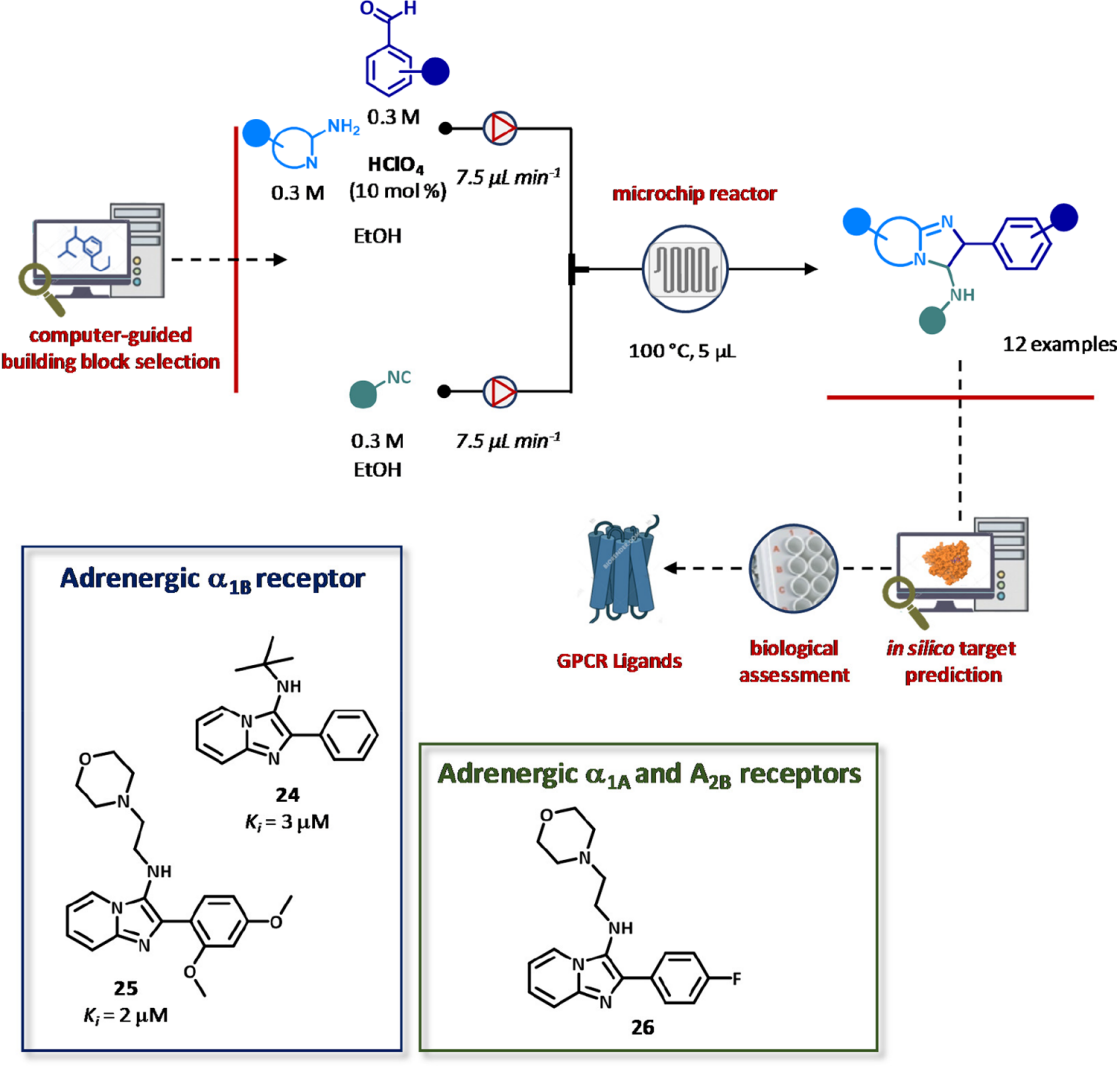
Microfluidic platform for the synthesis of imidazopyridines. Compounds were first screened by computer-based target prediction and best compounds were then tested for receptor activity. Desired products were prepared by acid-catalyzed Ugi three-component reaction between amine, aldehyde, and isocyanide and purified via preparative HPLC. Functional EC_50_ values obtained for the test compounds were converted to *K*_i_ values using the Cheng–Prusoff equation.

Automated fluidic platforms have also been coupled with de novo molecular design to drive building block selection for library expansion and hit optimization. A first example was reported by Schneider and co-workers at ETH (Zurich) by integrating microfluidic synthesis with computer-based target prediction for the rapid preparation and testing of imidazopyridine-based compounds (Figure 16).^122^ The Ugi three-component reaction was performed using a flow setup composed by a borosilicate DeanFlow chip with a total volume of 5 μL, a zigzag mixer, and a solenoid valve for automating the building block filling, dilution, and dispensing. After a preliminary screening of the reaction parameters, reactions were conducted by pumping a stock solution composed by amine (0.3 M), aldehyde (0.3 M), and 10% perchloric acid in ethanol and an ethanolic solution of the isocyanide (0.3 M) with a total flow rate of 15 μL min^–1^ (τ = 0.3 s, *T* = 100 °C). Thus, 12 imidazopyridines were generated with isolated yields ranging from 5% to 53% and ≥95% purity after preparative HPLC purification. The system was coupled with a Gaussian process regression model constructed from 469 known drug targets from ChEMBL database to obtain the predicted *p*Affinity against targets for all the compounds tested. As a result, five potential targets, including adenosine receptors A_1_ and A_2B_, adrenergic receptors α_1A_ and α_1B_, and PDE10A, were selected for further investigations. Radioligand displacement assays and cell-based functional activity assays led to identify nine out of 12 compounds that matched the predicted outcome from the regression model, with compounds **24** and **25** being active as antagonists at the adrenergic receptor α_1B_ (*K*_i_ = 2–3 μM) and compound **26** showing an antagonist profile for the adrenergic α_1A_ and adenosine A_2B_ receptors (Figure 16).

**Figure 17:**
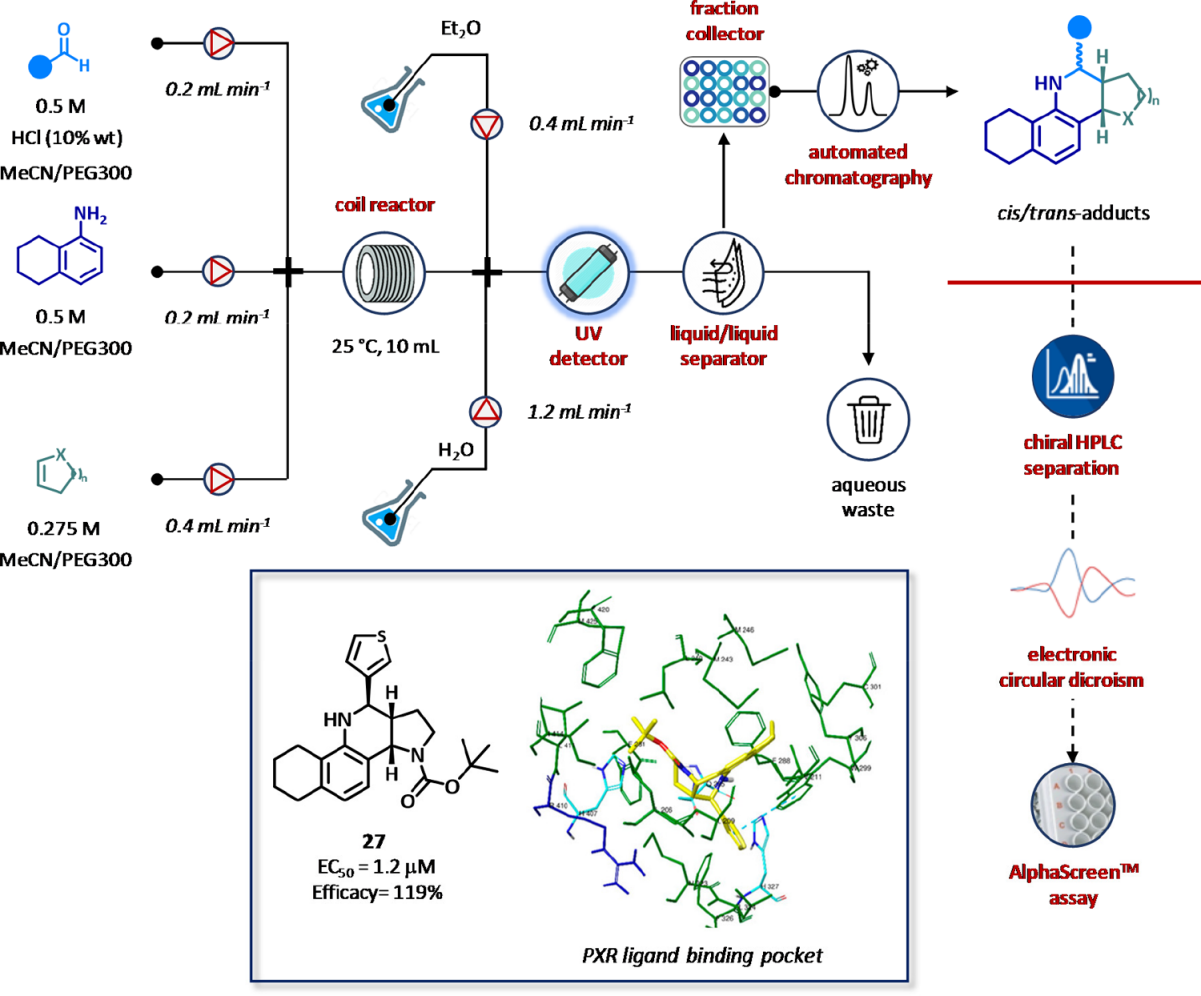
Automated flow synthesis, purification, and analysis tetracyclic tetrahydroquinolines as a novel class of PXR agonists. Products were obtained by multicomponent Povarov reaction, *cis*/*trans*-adducts were separated by flash chromatography, and single enantiomers were isolated by chiral HPLC and characterized by NMR and circular dichroism. Activity at the PXR receptor was determined by AlphaScreen technology.

We recently reported the integration of flow synthesizers, automation, analytical, and computational tools for the generation of chiral tetracyclic tetrahydroquinolines as novel Pregnane X Receptor (PXR) agonists (Figure 17).^123^ A collection of 29 analogues was rapidly synthesized by multicomponent Povarov reaction under automation and continuous flow conditions in three working days. Purification and stereochemical assignment of pure diastereoisomers was accomplished by a chiral-based HPLC method and in silico electronic circular dichroism (ECD) analysis realized by time-dependent density functional theory (TD-DFT) calculations. Pure isomers were then submitted to AlphaScreen assay to identify compound **27** as a low micromolar activator of PXR (EC_50_ = 1.2 μM) with an efficacy of 119%. Overall, the system stands to provide an ideal flow-based approach for the automated generation and characterization of multicomponent compound libraries for lead discovery and optimization. The proposed workflow can indeed be adopted for scaffolds featuring similar degrees of freedom and atoms to expedite medicinal chemistry and the development of stereoselective multicomponent methods (Figure 17).

## 4 End-to-End Machine-Assisted Discovery

Integrating automated flow synthesis with downstream operation, PAT, bioassays, and computing has the potential to reach optimal discovery cycle times. Closing the loop between synthesis, automation, drug design, and data analysis in small molecule drug discovery is gaining momentum, with diverse research groups having demonstrated the proof-of-concept as exemplified below.

**Figure 18:**
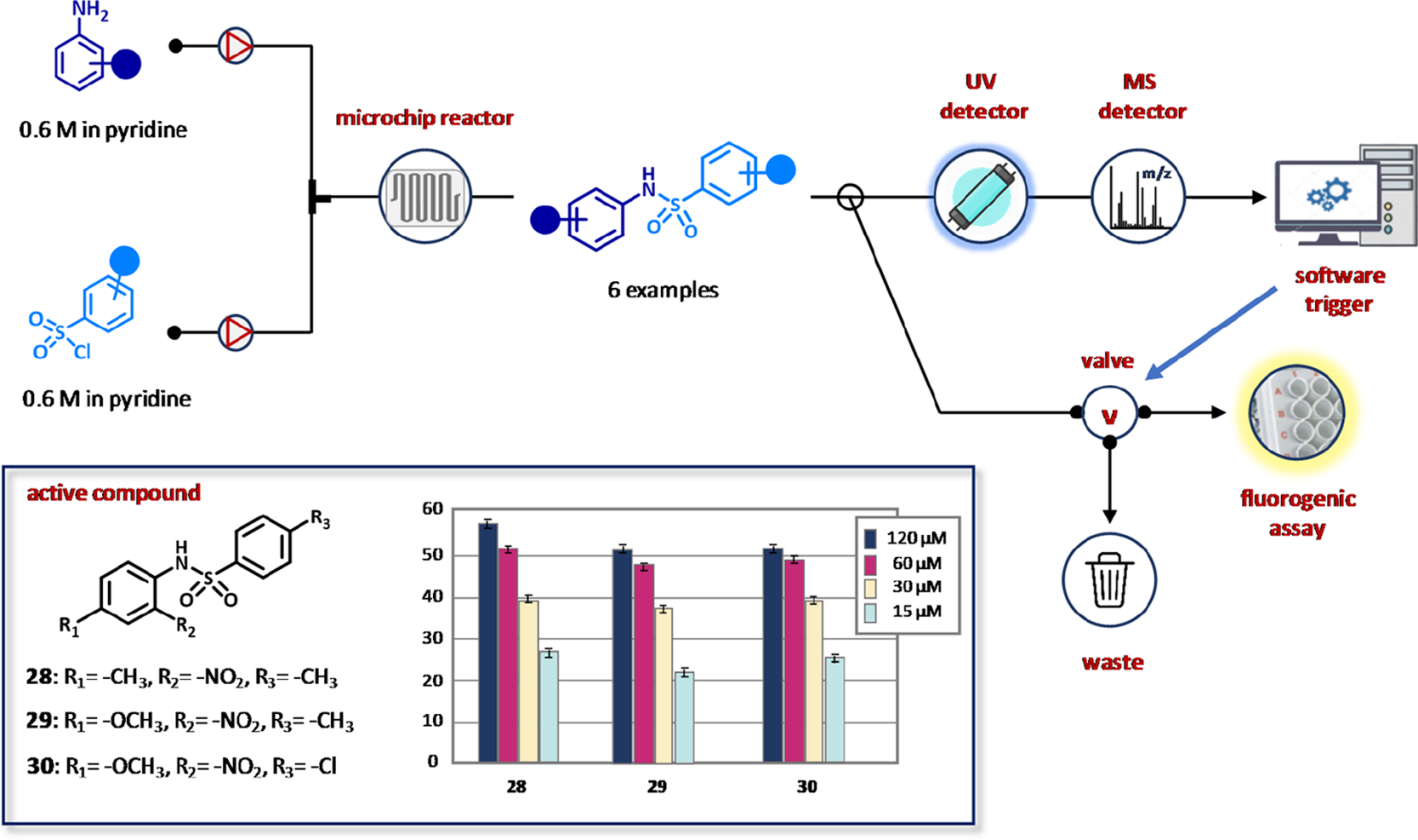
Integrated flow platform for the automated sulphonamide synthesis and in-line biological screening of T-cell tyrosine phosphatase (TCPTP) inhibitors. The synthesized compounds were tested using Caliper’s standard fluorogenic assay on Caliper 250 HTS system. The data were generated by monitoring the fluorescence of an on-chip incubation of TCPTP and 6,8-difluoro-4-methylumbelliferyl phosphate. Reproduced with permission from ref (124). Copyright 2005 Wiley.

A pioneering work aimed at creating a platform capable of integrating synthesis and biological screening was reported in 2005 by researchers at GlaxoSmithKline (Figure 18).^124^ A library of sulfonamides was prepared and continuously screened against T-cell tyrosine phosphatase (TCPTP). The equipment consisted of an UHPLC pumping system, a microchip reactor, an autosampler, a dilution device, a detection system, and a LC-MS for the analysis (Figure 18).

After the reaction of nitroanilines (0.6 M) with sulfonyl chlorides (0.6 M) in the presence of pyridine, products were passed through a LC column and splitted into two samples, one for UV/MS detection/analysis and the other for the in-line screening of the inhibitory effect on TCPTP by fluorogenic assays. Three out of six sulfonamides (**28**–**30**) exhibited up to 60% and 25% inhibition at 120 and 15 μM, respectively.

**Figure 19:**
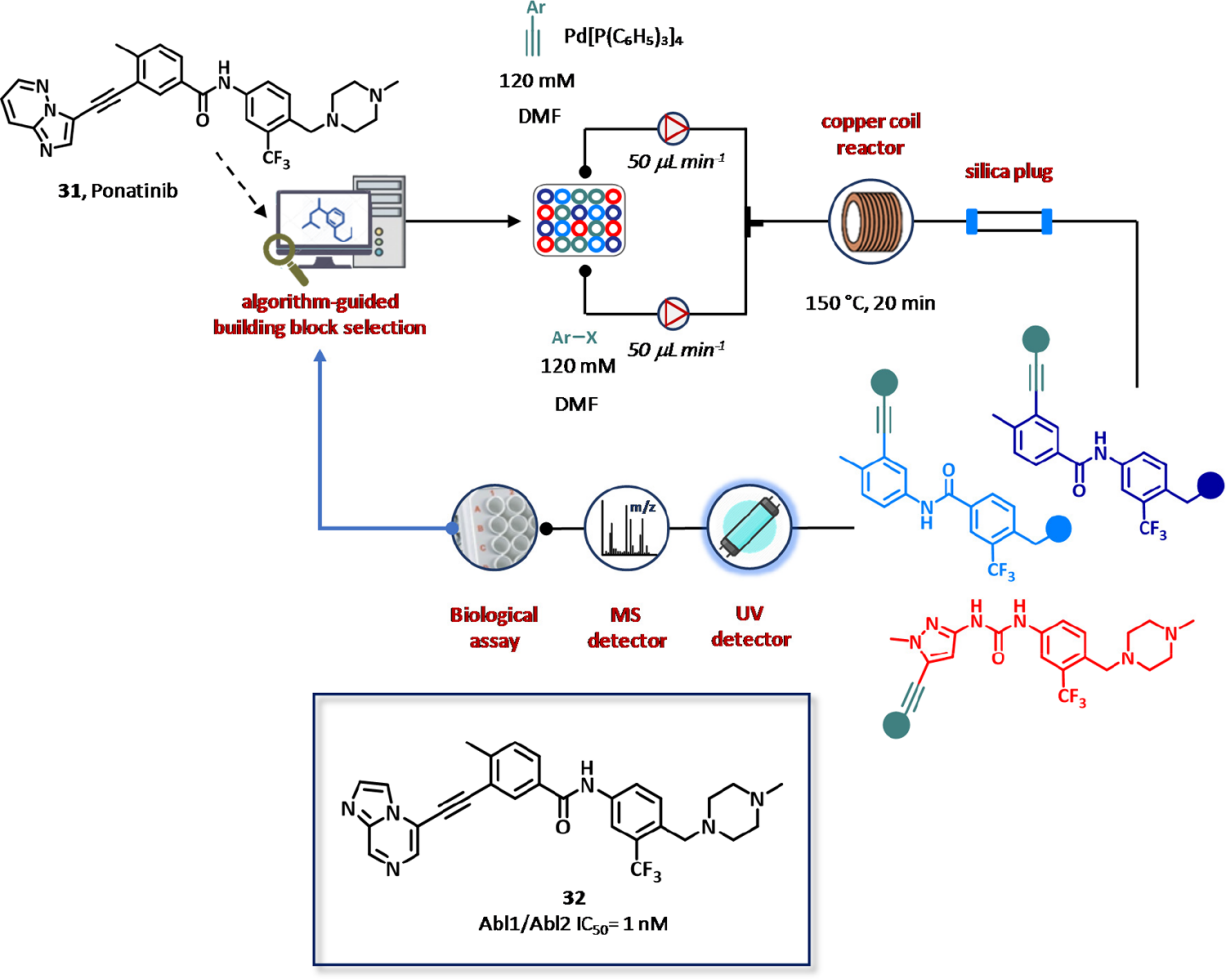
Closed-loop CyclOps platform from Cyclofluidic Ltd. for fully integrated and fully automated synthesis, purification, and screening assisted by algorithm-based drug design of Abl kinase inhibitors. Ponatinib analogues were synthesized by Sonogashira cross-coupling reaction between aryl halides and alkynes and purified by in-line preparative HPLC before testing. The Omnia kinase activity assay technology was employed to monitor the real-time kinase activity. For each tested inhibitor, a 3-fold dilution series was generated by an integrated liquid handling robot. The enzyme and the substrate solution were added to each test solution to assess the residual enzyme activity by fluorescence (excitation 360 nm, emission 485 nm). The data for each assay was fitted by linear regression and processed by Matlab software.

In 2013, Cyclofluidic Ltd., in collaboration with Sandexis LLP, Accelrys Ltd., and Sanofi-Aventis, designed a fully integrated autonomous platform assisted by an algorithm design (CyclOps) to streamline diverse hit-to-lead optimization programs (Figure 19).^125,126^ The idea was to provide a closed-loop SAR system able to integrate automation and continuous flow machines. CyclOps was composed by commercially available modular reactors equipped with a liquid handling element for delivering starting material and reagents into the injection loops. Once the reaction mixture eluted from the reactor, the crude mixture was purified by preparative HPLC and analyzed before being collected into a fraction collector. Once the final concentration and purity were assessed, an aliquot of the sample was taken by a liquid handling robot, diluted with the assay buffer, and tested in a chip-based fluorescence assay. The result obtained as IC_50_ value was readily processed by means of the design algorithm to suggest the next compound to be synthesized within the virtual space under investigation.

The platform was first validated for discovering novel Abl kinase inhibitors (Figure 19).^125^ Starting from X-ray diffraction (XRD) structure of Ponatinib (**31**), a known inhibitor that binds into the active site of Abl kinase, two structural hot-spots were identified and used for the design of ligands, which were prepared by Sonogashira cross-coupling reaction between aryl halides (10 fragments, 120 mM solution in DMF) and alkynes (27 fragments, 120 mM solution in DMF) in the presence of tetrakis(triphenylphosphine)palladium(0) as the catalyst using a copper coil as the flow reactor. In the first round of SAR generation, 22 compounds were generated and screened within the potential targeted chemical space (270 compounds) in only 30 h, generating a heat map matrix in which two algorithms assisted the molecular design of the next compound to be prepared. On the basis of the data obtained within the first experimental set, a second round of SAR cycle was next performed. After a total of 90 cycles of design–synthesis–screening, 64 new compounds were synthesized and evaluated against Abl1 and Abl2 kinase in approximately four days with an overall success synthetic rate of 71% and isolated yields ranging from 5% to 30%. From the study, 11 compounds emerged as potent inhibitors of Abl1/Abl2, with IC_50_ values in the low nanomolar range showing a high level of correlation with respect to conventional bioassay methods (Figure 19). Interestingly, the selected hit compounds were also potent inhibitors of all the clinically relevant Abl1 mutants and selective for Abl1 over P38α, with the amide derivative **32** being also endowed with a good membrane permeability and a suitable clearance in human liver microsomes.

**Figure 20:**
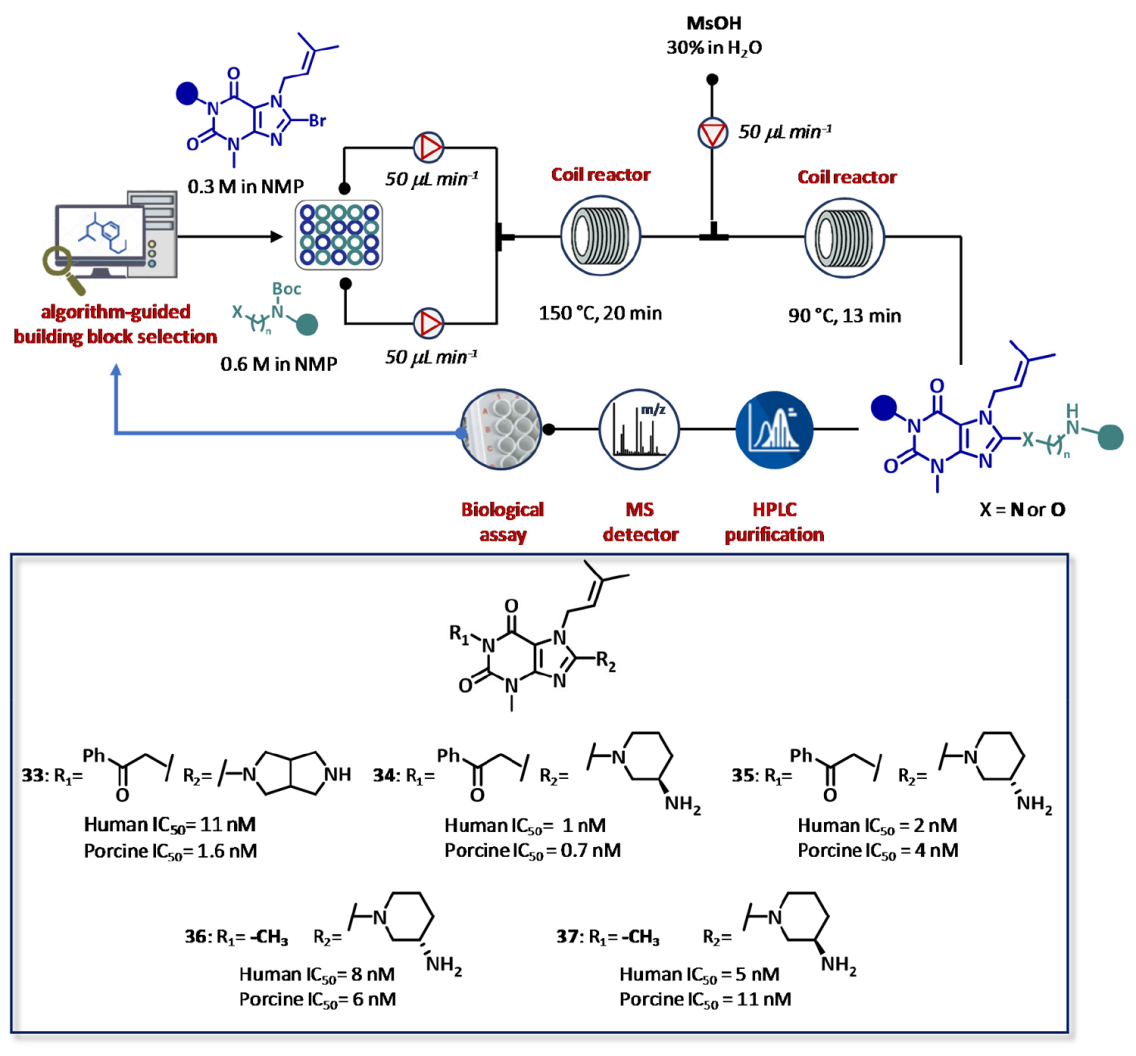
Closed-loop CyclOps platform from Cyclofluidic Ltd. for fully integrated and fully automated synthesis, purification, and screening assisted by algorithm-based drug design of dipeptidyl peptidase 4 (DPP4) inhibitors. The library was generated by nucleophilic substitution between BOC-protected diamines and 8-bromo xanthine derivatives, followed by acid-promoted deprotection of *tert*-butyloxycarbonyl group. After in-line purification by preparative HPLC, biological assays were carried out in 384 well plates. The enzyme (0.82 mU mL^–1^ for porcine DPP4 or 34 U mL^–1^ for human DPP4) was added, and the residual enzyme activity was monitored by adding the substrate at a final concentration equivalent to the *K*_M_^app^.

CyclOps was also employed for the development of xanthine-based dipeptidyl peptidase 4 (DPP4) inhibitors by a two-step automated flow synthesis (Figure 20).^126^ In particular, reactions were performed by automated injection of 8-bromosubstituted xanthine (0.3 M) in *N*-methyl-pyrrolidone (NMP) and a solution of the desired protected diamine (0.6 M) in the same solvent. After mixing, reactions took place in sequence in a 2 mL stainless steel coil heated at 150 °C with a total flow rate of 0.1 mL min^–1^ (τ = 20 min). The reactor outcome was then mixed with an aqueous solution of methanesulfonic acid (30 wt %) pumped at 50 μL min^–1^, and the mixture was pumped through a second 2 mL coil reactor heated at 90 °C (τ = 13 min) for BOC removal. The reaction products were purified by in-line LC–MS system, diluted with the assay buffer, and tested in a multiwell plate for determining the residual porcine and human DDP4 enzyme activity. Using this iterative cycle, 12 compounds were synthesized with yields ranging from 3% to 38% and tested in 24 h. Interestingly, five out of 12 molecules (compounds **33**–**37**) exhibited nanomolar inhibitory activity against both enzymes. The acquired data from SAR analysis were exploited to further extend the chemical exploration by using a series of amino alcohols instead of BOC-protected diamines. Overall, 29 compounds were prepared in high purity and tested in only three days with a chemistry success rate of 93%. Again, a high degree of correlation was observed with data previously obtained by traditional approaches (Figure 20).

**Figure 21:**
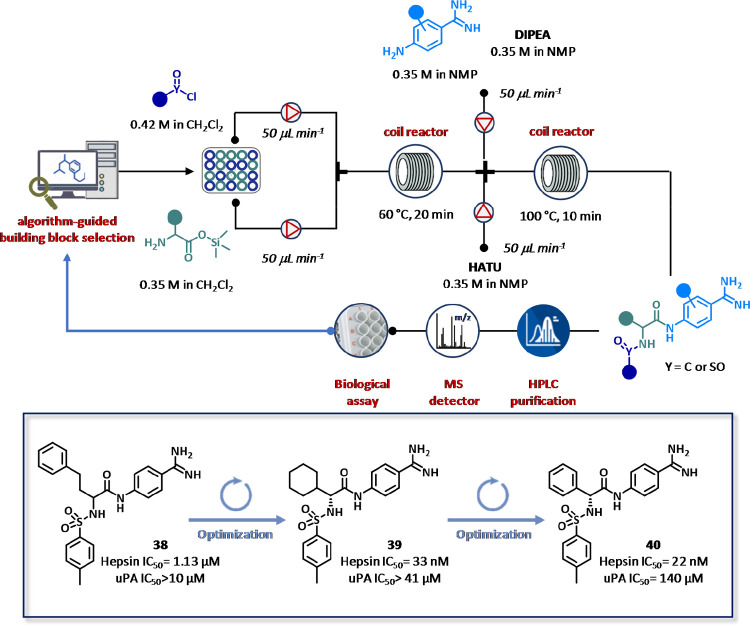
Closed-loop CyclOps platform from Cyclofluidic Ltd. for fully integrated and fully automated synthesis, purification, and screening assisted by algorithm-based drug design of hepsin inhibitors. Tested compounds were obtained by condensation between silylated amino acids and sulfonyl chlorides, acyl chlorides, or isocyanates, followed by amidation with amidine. The CyclOps bioassay module consisted of a fraction collection station, a reagent station, liquid handling robotics, plate store, an integrated plate reader, a syringe drive, and a two-way, six-port injection valve fitted with a 200 μL loop. Data thus generated were processed by CyclOps software and analyzed with Matlab suite for determining IC_50_ values.

Recently, Cyclofluidic has demonstrated the successful application of CyclOps platform in the field of hepsin inhibitors (Figure 21).^127^ In this case study, preliminary SAR data input generated from commercially available compounds were instrumental to identify a series of hits characterized by appreciable potency, selectivity, and chemical amenability for structural manipulations. In particular, starting from compound **38** (IC_50_ = 1.13 μM) endowed with good selectivity over urokinase-type plasminogen activator (uPA) (IC_50_ > 10 μM), a flow synthesizer was coupled with LC/MS/ELSD for analysis and purification, integrated with biological assays for hepsin and uPA test, and online chromatographic log *D* determination. The system was completed with an algorithm for substrate selection and potency prediction (Figure 21). In particular, stock solutions of silylated amino acid trimethylsilyl esters (0.35 M in CH_2_Cl_2_) were mixed with the desired sulfonyl chlorides, acyl chlorides, or isocyanates (0.42 M in CH_2_Cl_2_) in the presence of diisopropylethylamine (DIPEA) with a total flow rate of 100 μL min^–1^ and reacted into a 2 mL reactor coil at 60 °C. After 20 min, the intermediates were combined with a solution of amidine dihydrochloride and DIPEA (0.35 M in NMP) and a solution of hexafluorophosphate azabenzotriazole tetramethyl uronium (HATU) (0.35 M in NMP) employed as the coupling agent pumped with a flow rate of 50 μL min^–1^ for each pump. The mixture was reacted into a second 2 mL reactor coil (τ = 10 min) heated at 100 °C. A set of 63 reactants of amino acids, sulfonyl/acid chlorides/isocyanates, and aminoamidines was selected covering a virtual chemical space of 5472 compounds. A multiparameter optimization method, namely Best Objective Under-Sampled (BOUS), worked for designing new improved analogues. As a result, an initial set of 24 closed-loop synthesis and screening experiments was performed, furnishing a collection of 63 products with 70% of active compounds. This first SAR run allowed to reduce the virtual chemical space under investigation from 5472 to 297 chemical entities. The adoption of the Chase Objective tool for multiple closed-loop experiments led to the identification of **39**, a derivative containing (*R*)-cyclohexylglycine residue, as the most potent compound endowed with nanomolar IC_50_ value (33 nM) against hepsin and a selectivity index (hepsin/uPA) greater than 100. Two new sets of 22 synthesis-to-screen cycles and 21 closed-loops experiments were performed using a restricted subset of the amino acid pool. The (*R*)-phenylglycine derivative **40** emerged as the best compound being endowed with a nanomolar hepsin inhibition (IC_50_= 22 nM) and high uPA selectivity (>6000-fold). The lead **40** was characterized in terms of a selectivity profile against a panel of 10 serine proteases, ADMET profile (solubility, PAMPA permeability, metabolic stability in both mouse and human microsomes, cytotoxicity in Hep-G2 cells), and tested in oncogenic functional assays. Overall, the use of CyclOps platform provided 142 compounds (out of more than 5000 possible combinations) in nine days (90 min per cycle) with improved activity and selectivity over uPA.^100^

**Figure 22:**
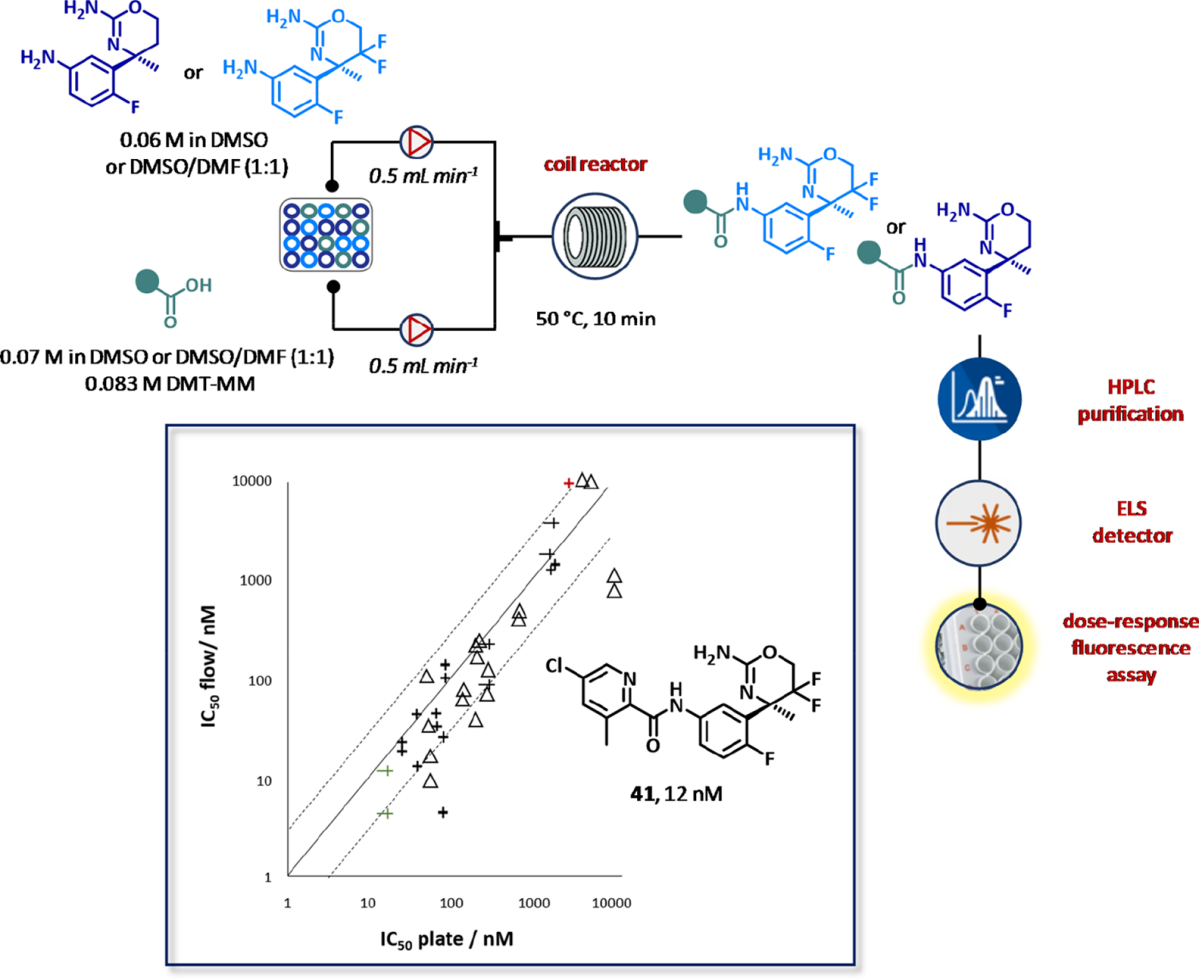
Fully integrated and automated flow system for the generation of BACE1 inhibitors. The bioassay chip was primed for 2 min with streams of enzyme (90 nM), substrate (0.9 μM), and assay buffer, at 0.8 mL min^–1^ for each pump. After in-line purification and analysis, each compound was injected into the dispersion capillary by a liquid handler directly from the preparative HPLC and dispersed by assay buffer into the chip. After 30 min of incubation time, using a gradient calibration, the enzyme activity was determined vs the corresponding fluoresce in concentrations, and the resulting dose–response curve was used to extrapolate the IC_50_ values. Reproduced with permission from ref (128). Copyright 2014 Wiley.

In 2014, researchers at Hoffman-LaRoche disclosed a similar autonomous assembled device for the rapid generation of β-secretase (BACE1) inhibitors.^128^ A small library of amides obtained from two commercially available anilines (0.06 M in DMSO or DMSO/DMF, 1:1) and 10 carboxylic acid synthons (0.07 M in DMSO or DMSO/DMF, 1:1) in the presence of 4-(4,6-dimethoxy-1,3,5-triazin-2-yl)-4-methylmorpholinium tetrafluoroborate (DMT-MM, 0.083 M in DMSO or DMSO/DMF, 1:1) was synthesized under flow conditions and purified via preparative HPLC (Figure 22). Aliquots (4–5 mL) of the purified compounds were collected by a liquid handler, analyzed by LC-MS for purity estimation (81–98%), quantified by a HPLC-ELSD calibrated method for assessing the final concentration, and tested in a dose–response chip-based assay to provide IC_50_ values within 60 min of total cycle time per compound. Interestingly, to improve sampling precision while minimizing the required amounts of compound for testing, a concentration gradient was generated by control diffusion using a glass capillary. Remarkably, the system produced reproducible SAR data that were comparable with those obtained by traditional methods. Compound **41** was finally identified as the most potent BACE1 of the library (IC_50_= 12 nM).

**Figure 23:**
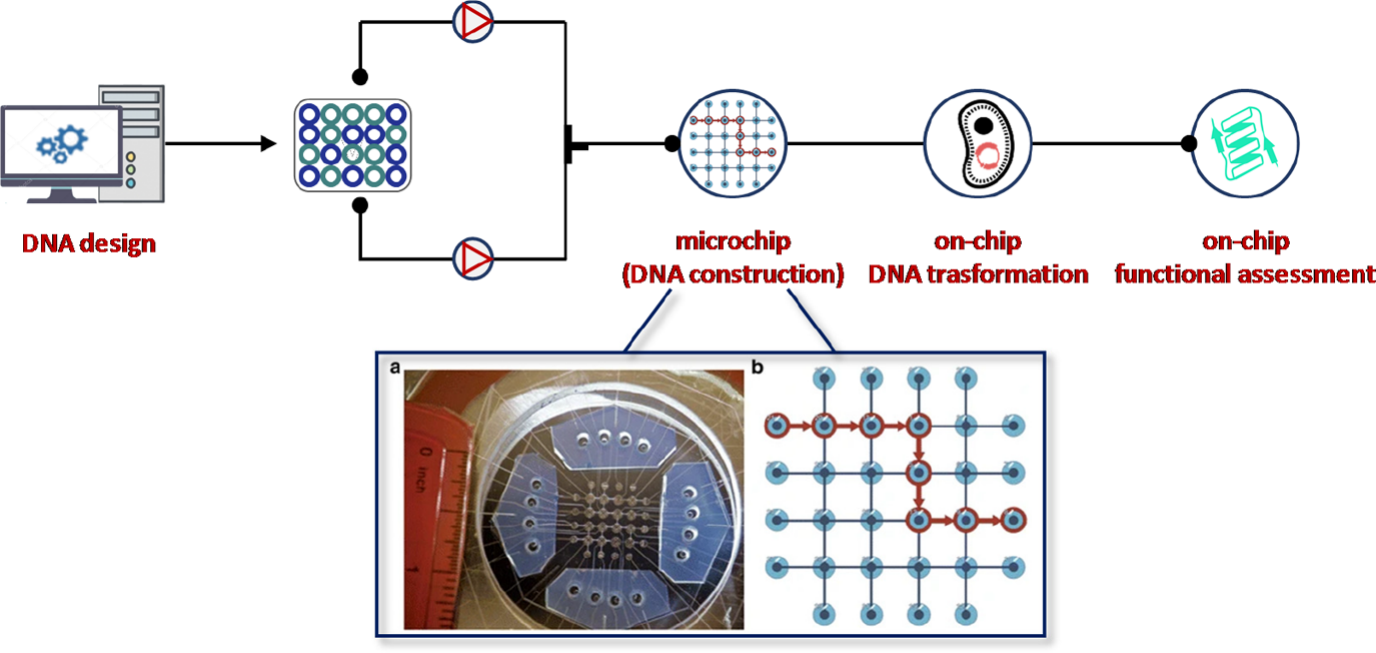
Schematic representation of the first automated microfluidic platform for synthetic biology.

In 2016, the first programmable and multipurpose microfluidic assembly for synthetic biology was developed.^129^ The system consisted of a microfluidic chip, an electronic pneumatic control system, a temperature regulator, and a software for automation with a web-based interface (Figure 23). This lab-on-chip platform was able to integrate and automate the iterative synthetic biology steps including the design of DNA libraries assisted by “DNA constructor” software, their synthesis, and transformation into different hosts (e.g.: *Escherichia coli* and *Saccharomyces cerevisiae*) by applying hierarchical DNA construction (IHDC). Such an approach was specifically designed for microfluidic functional assays, including cell growth, protein expression induction, and colorimetric assay as well as image data analysis.

**Figure 24:**
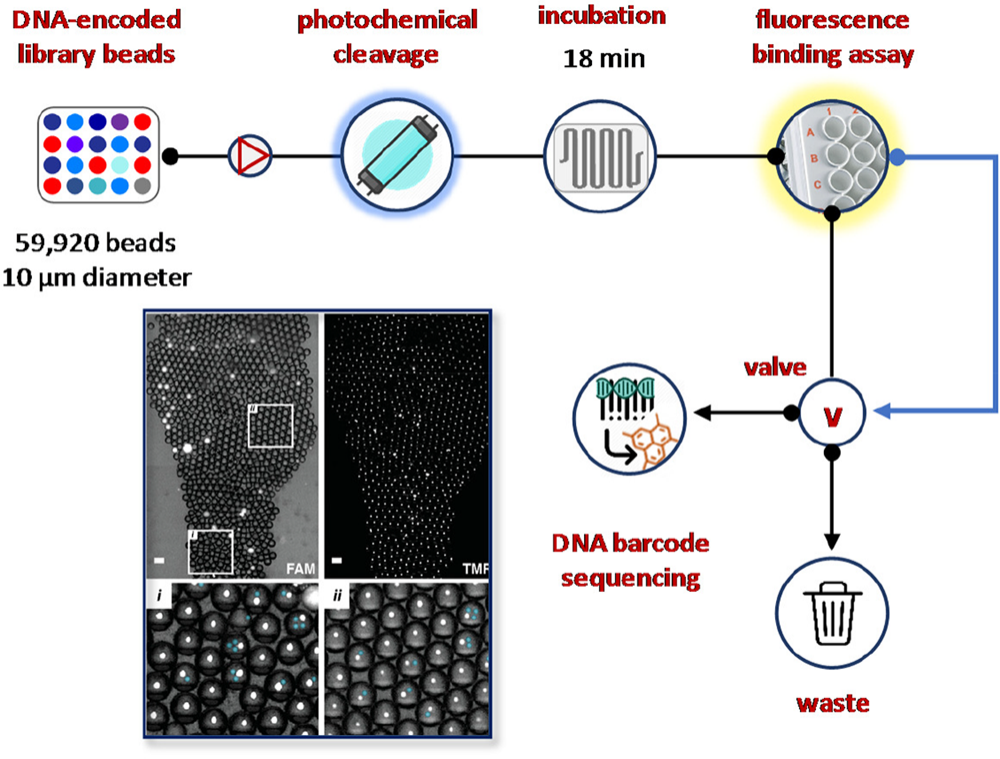
Microfluidic platform for ultrahigh-throughput hit deconvolution by sequencing of DNA-encoded compound beads. An integrated waveguide irradiates the droplet flow at 365, inducing the photochemical cleavage of compound from the bead into the droplet volume. Droplets dosed with compound (1–3 μM) are then incubated for 18 min within a Frenz-type delay line, at the end of which droplets are focused back into single file and the confocal laser-induced fluorescence detectors measure droplet fluorescence and the software analyze the data.

In analogy to this work, MacConnell and co-workers described the development of a microfluidic-based apparatus for ultrahigh throughput hit deconvolution by sequencing (Figure 24).^130^ The miniaturized device was able to screen DNA-encoded compound beads by carrying out library bead distribution into pL-scale assay reagent droplets, photochemical cleavage of compound from the bead, assay incubation, laser-induced fluorescence-based binding assay for hit identification, and isolation by means of fluorescence-activated droplet sorting and DNA barcode sequencing. Thus, DNA-encoded beads (10 μm diameter, 1920 beads, 729 encoding sequences) endowed with a positive control inhibitor pepstatin A were mixed with negative control beads (58000 beads, 1728 encoding sequences) and screened for cathepsin D inhibition using a biochemical enzyme activity assay. Overall, the screening required only 120 μL of mixed assay volume and 0.05 mg of library beads within 4 h of assay (18 min incubation over 240 min analysis). Remarkably, by applying the template barcoding strategy, it was possible to reduce the false discovery rate to 2.6% compared to 24% obtained by visual inspection.

**Figure 25:**
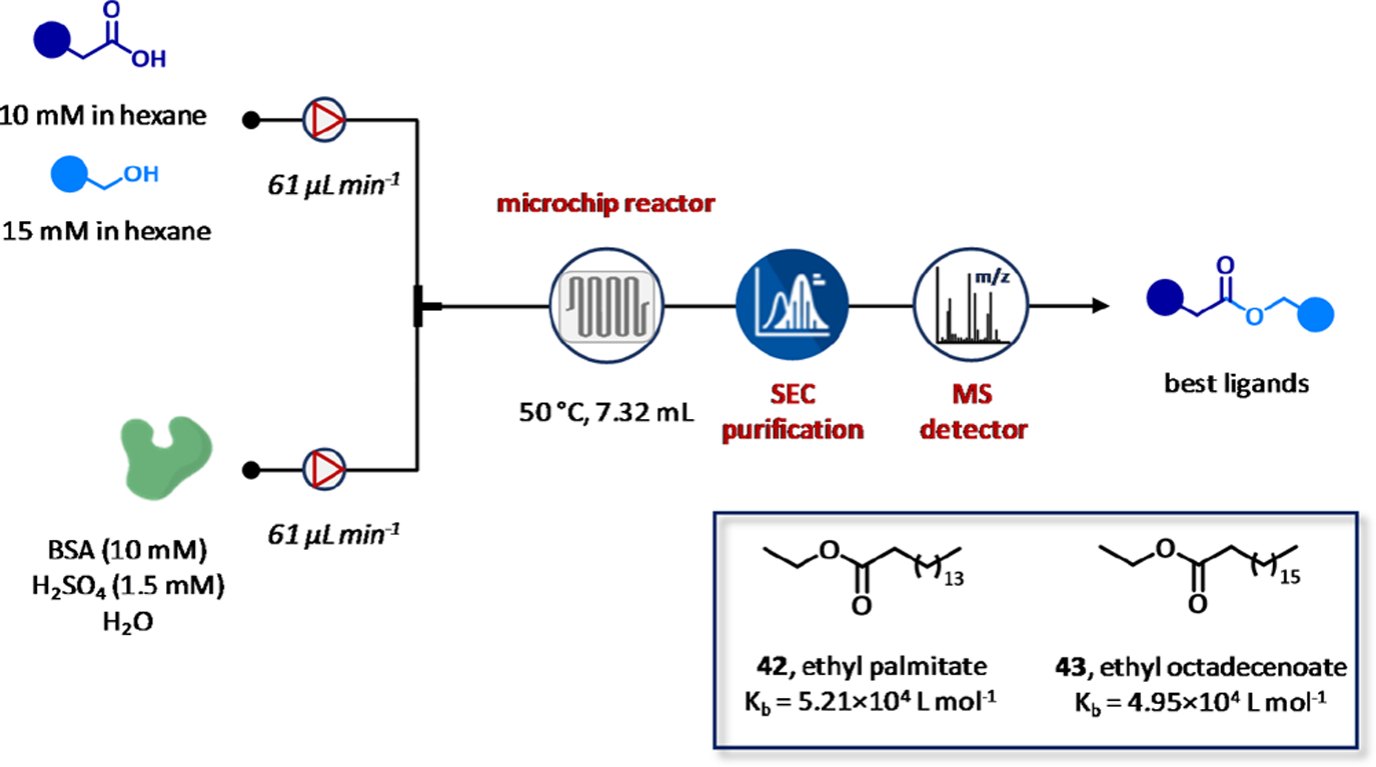
Integrated flow platform for the synthesis and identification of protein binders. Fluorescence spectra for each compound were recorded at concentration ranging from 0.4 to 2.4 nM, *T* = 298 K, pH = 7.4, and λ_emission_ = 280 nm.

Recently, Guo and co-workers have reported an integrated platform for the flow-based synthesis and identification of protein binders (Figure 25).^131^ In particular, by using a rapid microscale flow synthesis coupled with size-exclusion chromatography (SEC)-MS technology, the modular platform solved the main limitations of the conventional protein-directed dynamic combinatorial chemistry (DCC), including the poor reactivity of inhibitors at low concentrations, the reduction of protein activity or decomposition of inhibitors for long equilibration times, as well as the low throughput of currently available analytical detection methods. This system was applied to the identification of competitive inhibitors of bovine serum albumin (BSA) by preparing and testing dynamic combinatorial libraries (DCLs) based on the principle of fragment-based drug discovery from a pool of alcohols and carboxylic acids. In particular, first, a small DCL of four members was synthesized both under microfluidic and conventional batch conditions. The setup involved the esterification of the desired carboxylic acid (10 mM) and alcohol (15 mM) in hexane. The resulting DLCs members were pumped at 61 μL min^–1^ of flow rate and combined with a stream of H_2_SO_4_ (pH = 1, 1.5 mM) and BSA (10 mM) in distilled water pumped at 61 μL min^–1^ of flow rate. Incubation was conducted in a microreactor (*V* = 7.32 mL, τ = 60 min) at 50 °C using a GC detector to monitor the reaching of the equilibrium. It is worth noting that the higher surface area-to-volume ratio and the enhanced mass/heat transfer of microfluidic over batch modality resulted in 12-fold reduction of the equilibration time. The versatility of this approach was evaluated by preparing a larger DCL composed by 528 members. Interestingly, while HRMS analysis of DCL prepared under batch modality detected the already known ethyl palmitate **42** as the sole binder of BSA, the microfluidic platform allowed discovery of ethyl octadecanoate **43**. At this point, a fluorescent assay was carried out to confirm the binding of the ethyl octadecanoate **43**. As a result, a concentration-dependent quenching of the intrinsic fluorescence of BSA (excitation at 280 nm and fluorescence emission at 348 nm) was observed with binding constants *K*_D_ of 4.95 and 5.21 × 10^4^ L mol^–1^ for ethyl octadecanoate **43** and ethyl palmitate **42**, respectively. Finally, the results obtained from the quantitative analysis of the binding of ethyl octadecanoate **43** to BSA performed by Stern–Volmer dynamic quenching assay at different temperatures confirmed the involvement of only one binding site in the formation of the complex BSA–ethyl octadecanoate.

## 5 Conclusions and Future Perspectives

“I’ve done nothing but spend money the entire time” said the medicinal chemist Dr. Derek Lowe, as reported by Molly Ferguson for STAT news.^132^ Beyond being a common feeling of many medicinal chemists, the words of Dr. Lowe emphasize how much failure goes into drug discovery. Although failures are part of the process and may indirectly contribute to the discovery of novel drugs, they come at a cost in terms of competitiveness and efficiency. Substantial improvements demand abilities and creativity in finding tailored solutions by adopting new concepts and adjusted strategies for future.^7,16,133^ Advances in science and technology such as synthesis machines, activity prediction models, and automated screening facilities can tremendously revolutionize drug discovery, especially at the early stages, offering a fertile ground of interest in both academia and pharmaceutical companies to validate innovative approaches and their translational potential.^134^

In this framework, medicinal chemistry is often viewed as a limiting step being traditionally based on discontinuous and compartmentalized cycles of “design–synthesis–test–analysis” that make the process slow and expensive in terms of human and economic resources. Among compartments, organic synthesis is the most time- and resource-consuming stage and often determines the number of compounds that enters into clinical trials. While the question over the real advantage of using robot-drug discoverers is still under debate,^135^ recent advances in integrative technologies have demonstrated to concretely address some of the current limitations of medicinal chemistry. Evolution from handmade chemistry to automated synthesizers and their integration with molecular design, PAT and in line testing can usher in a whole new era of drug discovery, thereby accelerating the journey from hit to marketed drug. However, it is important to not repeat past mistakes in overestimating the potential of technologies but rather to critically analyze benefits and drawbacks at the current state-of-the-art. While the automated generation of chemical libraries and the systematic optimization of promising leads by adapting learning cycles can be considered largely a fait accompli, the realization of fully integrated platforms for compound design, synthesis, assay, and data analysis is still challenging and far from laboratory routine. Although encouraging progresses have been made as we have highlighted in this Perspective, their widespread application needs to be proved. Reasons for reluctance in adoption of these approaches, particularly by industry, may be due to a lack of awareness or confidence in such methods. To this aim, scientists across the various disciplines, management, and investors need to share the goals and efforts to ensure the aforementioned technologies demonstrate utility in a medicinal chemistry and drug discovery context. Only after effective implementation, skepticism around machine-mediated discovery can be erased.

In this evolving scenario, academics have to restructure education and research to reduce the large gap between basic science, translational research, and drug development (the “valley of death”)^134^ while improving the quality of collaborations between academia and industry that ultimately have to drive toward innovation. Technology will certainly play an increasingly important role in research and development, and the hope is to become an indispensable part of the future education in chemistry and neighboring disciplines. While chemists will continue to develop new and improved method for synthesis, attention should be also directed to the tremendous opportunity of adopting technological solutions. We foresee the future medicinal chemistry and early drug discovery based on the ideal combination of human skills and creativity, automated machines, and AI. We firmly believe that only through an increasing collaboration between academia and pharmaceutical companies, as well as financial institutions and government agencies, future challenges can be tackled with a profound impact on chemical sciences, drug discovery research, and finally on human health.

## Abbreviations Used

AI: artificial Intelligence
BOUS: best objective under-sampled
CAD: computer-aided design
CAI: chemical artificial intelligence
CFITA: continuous-flow injection titration assay
CombiChem: combinatorial chemistry
CPP: critical process parameters
CQA: critical quality attributes
DCC: dynamic combinatorial chemistry
DCLs: dynamic combinatorial libraries
DOS: diverse oriented synthesis
ECD: electronic circular dichroism
FAC: frontal affinity chromatography
FEP: fluorinated ethylene propylene
HTE: high-throughput experimentation
IHDC: hierarchical DNA construction
IoT: Internet of Things
LabVIEW: Laboratory Virtual Instrumentation Engineering Workbench
ML: learning machines
PAMPA: parallel artificial membrane permeability assay
PAT: process analytical technology
PFA: perfluoroalkoxy alkane
PTFE: polytetrafluoroethylene
QbD: quality-by-design
SEC: size-exclusion chromatography
SNOBFIT: stable noisy optimization by branch and fit
SWIFT: synthesis with integrated flow technology
TCP/IP: transmission control protocol/internet protocol
TD-DFT: time-dependent density functional theory
XRD: X-ray diffraction
